# Diets and Cellular-Derived Microparticles: Weighing a Plausible Link With Cerebral Small Vessel Disease

**DOI:** 10.3389/fcvm.2021.632131

**Published:** 2021-02-24

**Authors:** Che Mohd Nasril Che Mohd Nassir, Mazira Mohamad Ghazali, Sabarisah Hashim, Nur Suhaila Idris, Lee Si Yuen, Wong Jia Hui, Haziq Hazman Norman, Chuang Huei Gau, Nanthini Jayabalan, Yuri Na, Linqing Feng, Lin Kooi Ong, Hafizah Abdul Hamid, Haja Nazeer Ahamed, Muzaimi Mustapha

**Affiliations:** ^1^Department of Neurosciences, School of Medical Sciences, Universiti Sains Malaysia, Kubang Kerian, Malaysia; ^2^Department of Family Medicine, School of Medical Sciences, Universiti Sains Malaysia, Kubang Kerian, Malaysia; ^3^Department of Internal Medicine, School of Medical Sciences, Universiti Sains Malaysia, Kubang Kerian, Malaysia; ^4^Neurobiology of Aging and Disease Laboratory, Lee Kong Chian School of Medicine, Nanyang Technological University, Singapore, Singapore; ^5^Anatomy Unit, International Medical School (IMS), Management and Science University (MSU), Shah Alam, Malaysia; ^6^Department of Psychology and Counselling, Faculty of Arts and Social Science, Universiti Tunku Abdul Rahman (UTAR), Kampar, Malaysia; ^7^Translational Neuroscience Lab, University of Queensland (UQ), Centre for Clinical Research, The University of Queensland, Herston, QLD, Australia; ^8^Center for Functional Connectomics, Brain Science Institute, Korea Institute of Science and Technology (KIST), Seoul, South Korea; ^9^School of Pharmacy, Monash University Malaysia, Bandar Sunway, Malaysia; ^10^School of Biomedical Sciences and Pharmacy, Priority Research Centre for Stroke and Brain Injury, University of Newcastle, Callaghan, NSW, Australia; ^11^Hunter Medical Research Institute, New Lambton Heights, NSW, Australia; ^12^Centre of Research Excellence Stroke Rehabilitation and Brain Recovery, National Health and Medical Research Council (NHMRC), Heidelberg, VIC, Australia; ^13^Department of Human Anatomy, Faculty of Medicine and Health Sciences, Universiti Putra Malaysia, Serdang, Malaysia; ^14^Crescent School of Pharmacy, B.S. Abdur Rahman Crescent Institute of Science and Technology, Chennai, India; ^15^Hospital Universiti Sains Malaysia, Jalan Raja Perempuan Zainab II, Kubang Kerian, Malaysia

**Keywords:** cerebral small vessel disease, diet, microparticles, neurodegeneration, microthrombosis

## Abstract

Cerebral small vessel disease (CSVD) represents a spectrum of pathological processes of various etiologies affecting the brain microcirculation that can trigger neuroinflammation and the subsequent neurodegenerative cascade. Prevalent with aging, CSVD is a recognized risk factor for stroke, vascular dementia, Alzheimer disease, and Parkinson disease. Despite being the most common neurodegenerative condition with cerebrocardiovascular axis, understanding about it remains poor. Interestingly, modifiable risk factors such as unhealthy diet including high intake of processed food, high-fat foods, and animal by-products are known to influence the non-neural peripheral events, such as in the gastrointestinal tract and cardiovascular stress through cellular inflammation and oxidation. One key outcome from such events, among others, includes the cellular activations that lead to elevated levels of endogenous cellular-derived circulating microparticles (MPs). MPs can be produced from various cellular origins including leukocytes, platelets, endothelial cells, microbiota, and microglia. MPs could act as microthrombogenic procoagulant that served as a plausible culprit for the vulnerable end-artery microcirculation in the brain as the end-organ leading to CSVD manifestations. However, little attention has been paid on the potential role of MPs in the onset and progression of CSVD spectrum. Corroboratively, the formation of MPs is known to be influenced by diet-induced cellular stress. Thus, this review aims to appraise the body of evidence on the dietary-related impacts on circulating MPs from non-neural peripheral origins that could serve as a plausible microthrombosis in CSVD manifestation as a precursor of neurodegeneration. Here, we elaborate on the pathomechanical features of MPs in health and disease states; relevance of dietary patterns on MP release; preclinical studies pertaining to diet-based MPs contribution to disease; MP level as putative surrogates for early disease biomarkers; and lastly, the potential of MPs manipulation with diet-based approach as a novel preventive measure for CSVD in an aging society worldwide.

## Introduction

An acute cerebrovascular event due to an occlusion (or ischemia) of small blood vessels deep within the brain is a known manifestation of small vessel disease (SVD) involving the brain small end arteries, capillaries, venules, and arterioles (1–3). Of all ischemic stroke events, ~30% are represented by cerebral SVD (CSVD) (1, 4). CSVD is a spectrum of complex and overlapping pathophysiological mechanism of various etiologies affecting the brain small vessel microcirculation that can trigger neuronal inflammation and the subsequent neurodegenerative cascade. However, it is generally viewed that CSVD represents pathological consequences of SVD on the brain parenchyma rather than the underlying diseases of the vessels (5). Prevalent with aging, CSVD is recognized as risk factor for stroke, vascular dementia, Alzheimer disease (AD), and Parkinson disease (PD) (6, 7). Despite being arguably the most common neurodegenerative disease (NDD) with predilection of the cardiocerebrovascular axis, there is only limited knowledge about CSVD underlying mechanisms.

Among the known modifiable risk factors for stroke, dietary patterns are recognized to modulate the non-neural peripheral events such as in the gastrointestinal tract (GIT) (i.e., GIT dysbiosis) and cardiovascular stress through cellular inflammations and oxidation. Moreover, diet plays a crucial role in maintaining the physiological systems responsible for homeostasis and hemostasis, whereby healthy dietary pattern has been classified as diet with lower concentration of plasma proinflammatory markers (8). Hence, certain dietary patterns could potentially lead to undesirable alterations in such systems as shown in the case of less or non-nutritious/unbalanced diets (9, 10). Moreover, unhealthy dietary habits have been reported to contribute to higher risk of developing metabolic disease, coronary heart disease, and stroke (11) and likely to modulate systemic peripheral events that can influence the development and progression of NDD such as CSVD. One key outcome from such events, among others, includes the cellular activations that lead to elevated levels of endogenous cellular-derived circulating microparticles (MPs). MPs can be produced from various cellular origins including leukocytes, platelets, endothelial cells (ECs), microbiota, and microglia. MPs could act as microthrombogenic procoagulant that could be detrimental to the vulnerable microcirculation, particularly the penetrating, poorly collateralized end-arteries in the brain parenchyma, leading to CSVD manifestations. However, little attention has been paid on the potential role of MPs in the onset and progression of CSVD spectrum. Corroboratively, the formation of MPs is known to be influenced by diet-induced cellular stress.

Thus, this review aims to appraise the body of evidence on the dietary-related impacts on circulating MPs from non-neural peripheral origins that could serve as a plausible microthrombogenic role in CSVD manifestation and hence a precursor of NDD. Here, we elaborate on the pathomechanical features of MPs in health and disease states; relevance of dietary patterns on MP release; preclinical studies pertaining to diet-based MPs contribution to disease; MP level as putative surrogates for early disease biomarkers; and lastly, the potential of MPs manipulation with diet-based approach as a novel preventive measure for CSVD.

## Microcirculation Network and Small Vessel Disease

**Figure 1:**
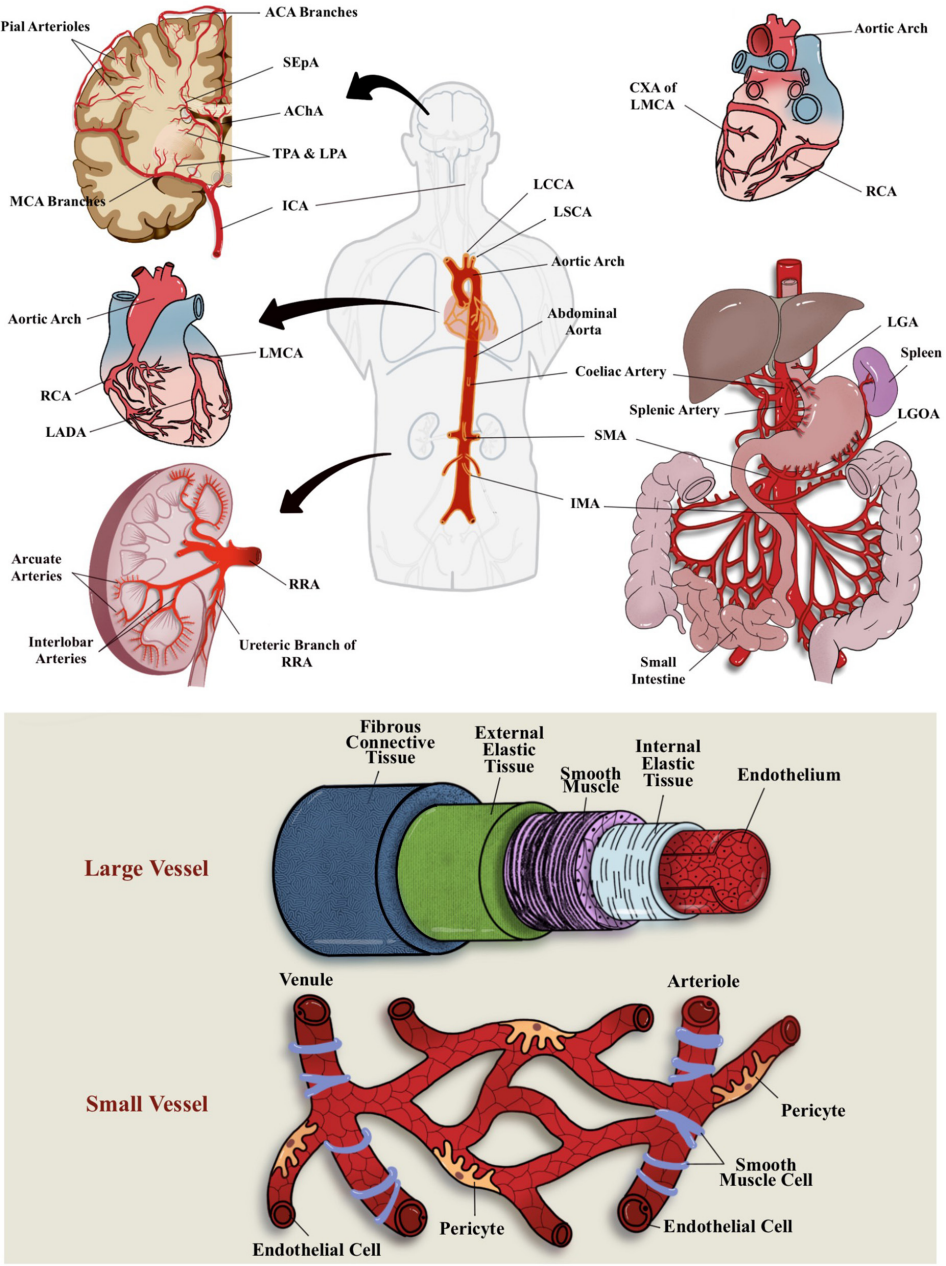
Vascular blood (arterial) supplies to the brain, heart, GIT, and kidney and differential structure between large and small vessel. Most of these organs receives their blood supply locally or from the abdominal aorta. ACA, anterior cerebral arteries; AChA, anterior choroidal arteries; CXA, circumflex artery; ICA, internal carotid artery; IMA, inferior mesenteric arteries; LADA, left anterior descending artery; LCCA, left common carotid artery; LGA, left gastric artery; LGOA, left gastro-omental artery; LMCA, left main coronary artery; LPA; lenticulostriate perforating arteries; LSCA, left subclavian artery; MCA, middle cerebral artery; RCA, right coronary artery; RRA, right renal artery; SEpA, subependymal arteries; SMA, superior mesenteric artery; TPA, thalamic perforating arteries.

The term *microcirculation* used to represent the terminal vascular branches or network of the systemic circulation that consist mainly of (small) microvessel (diameters of <20 μm) (12). These microvessels comprised capillaries (including their subcellular components), arterioles, and postcapillary venules (13) (Figure 1). For example, in coronary blood supply (i.e., from right coronary artery, right coronary artery, and left main coronary artery), small muscular arteries are found throughout the myocardium that further branch into an extensive capillary bed (intramural arteries) that embraces the cardiac myocytes (14). In GIT, the small perforating arteries mainly originated from celiac trunk (arteries) that supply the foregut (i.e., esophagus, stomach, liver, gallbladder, superior pancreas, first and second part of duodenum), superior mesenteric artery supply the midgut (i.e., third part of duodenum, jejunum, appendix, cecum, ascending colon), and inferior mesenteric arteries that supply the hindgut (i.e., descending colon, rectum, upper part of anal canal) (15, 16). While renal microvasculature are smaller branches that form the afferent arterioles leading to the formation of glomerular capillaries, the distal glomerular capillaries form the efferent arterioles, followed by the peritubular capillaries that supply the renal tubules (17). In the brain, ~72% of cerebral blood flow (cBF) is contributed by anterior circulation that arises from the internal carotid artery (ICA) (18). cBF can be defined as the volume of blood that flows per unit mass per unit time in brain tissue [mL_blood_/(100 g_tissue_ min)], or flow per unit volume of brain tissue [mL_blood_/(100 mL_tissue_ min)] (19). From among the vast ICA branching network, the most significant pathophysiologically are the anterior cerebral arteries, middle cerebral arteries (MCAs), and anterior choroidal arteries. The branches of these arteries mainly supply the forebrain (i.e., frontal, temporal, and parietal lobes), as well as subcortical region of diencephalon and internal capsule. In addition, ~30% of cBF is contributed by posterior cerebral circulation that is derived from tributaries of the vertebral and basilar arteries (13). These branches mainly supply posterior portion of brain, i.e., occipital lobes and posterior brainstems (see Figure 1 for the illustration of blood supply to these major organs).

Pertaining the connection of vascular supply and drainage between these major organs, most of these organs receive their blood supplies locally or from the abdominal aorta (Figure 1). For example, some parts of the large intestine receive blood supply from the SMA (branching of the abdominal aorta) (16). The heart, on the other hand, consists of its own coronary vascular supply for oxygenated blood and coronary sinus for its venous drainage (14). All in all, most organs return deoxygenated blood either through superior or inferior vena cava for gaseous exchange through the pulmonary circulations. A direct connection between organs, for example, GIT–heart–brain axis, may be observed through the venous drainage but not through the arterial blood supply, whereby most of the GIT (visceral organs) circulation will return to inferior vena cava of the heart *via* the hepatic portal circulation (16). As for the cerebral circulation, the venous drainage will eventually reach the superior vena cava of the heart and, subsequently, the pulmonary circulation for gaseous exchange. For the brain, oxygen and nutrients from peripheral circulation are delivered through MCAs and their fenestrated capillaries that supply deep subcortical region (20). Hence, any initial peripheral event (from systemic and cellular insult or activation) may affect a specific organ through their local circulation or may even propagate *via* the abdominal aorta to other specific organ locations. Similarly, vascular drainage that eventually returns the deoxygenated blood from other organs of the body to the superior and inferior vena cava of the heart may also act as a “hitchhike” passageway for the systemic or cellular insults or activation by-products and lodged to other organs or blood vessels, including microcirculation network.

Consequently, microcirculation network is the most crucial compartment and terminal destination of the vascular systems, whereby it is the pinnacle site where the red blood cells (RBCs) in the capillaries directly transfer the oxygen to the surrounding parenchymal cells that require oxygen for energy metabolism (12). Apart from that, microcirculation helps to regulate intravascular-tissular space solute exchange, transporting all the nutrients and blood-borne hormones to the cells and tissues and moderating the functional activities of hemostasis and immune system (12). The vasculature of microcirculation consists primarily of lining of the ECs. The morphology and density of these endothelial structures varied between organs and vessels. However, endothelial lining generally consists of pores and fenestration that are held together by various adherent molecules such as cadherins and gap junctions (to carry current), hence allowing upstream electrical communication (12). Furthermore, ECs are symbiont with smooth muscle cells (SMCs) regulating the microvascular blood flow through the regulation of arteriolar vasotone with three different mechanisms, i.e., metabolic, myogenic, and neurohumoral control. The lumen of endothelium consists of gel-like structure (0.2–0.5 μm) synthesized by ECs, known as glycocalyx (e.g., proteoglycans, glycosaminoglycans, and plasma protein), which help in mediating endothelium functions, i.e., their microcirculatory functions (21, 22). Apart from glycocalyx, various subcellular substances are also present in the lumen of endothelium such as superoxide dismutase and antithrombin (23).

Therefore, the integrity of microvessel endothelium and its component is the main determinant for vascular barrier. Endothelial dysfunction is one of the ultimate cellular events that are responsible for hemodynamic changes seen in various pathological conditions (22). Microcirculation network is crucial for normal functioning of GIT, heart, and the nervous system, with the majority (up to 80%) of oxygen supplies to these organs is utilized for adenosine triphosphate production to aid sodium and potassium pumps maintaining the homeostasis. Thus, oxidative stress, hypoxia, nitro stress, and inflammatory mediators could potentiate the sequelae that lead to various SVD of these organs (24). Preclinical studies (including animal models) had shown that microcirculation and endothelial inflammation may serve as therapeutic targets to arrest microvascular-based organ or parenchymal injury (25, 26).

### Small Vessel Disease—An Overview

SVD is a term used to represent the pathological process that damages the small end arteries, capillaries, venules, and arterioles (2). The condition may lead to alteration of microcirculation (i.e., blood flow or perfusion) of the affected organ. SVD is generally observed in major organs such as the brain, retinal, heart, and urinary system (i.e., kidney), due to fact that these organs primarily required a desirable amount of cardiac output for their functionality (27). However, the GIT arteries are rarely affected to vascular disease either SVD or large vessel disease (i.e., atherosclerosis) (28). In rare instances, especially following myocardial infarction or atrial fibrillation, thrombus may accumulate and cause occlusion in the artery resulted in ischemic colitis (with an acute onset of abdominal pain and blood in the stools) (28, 29). Moreover, the thrombus or arterial occlusions may cause the reduction of blood flow (chronically) in the colon that can trigger inflammation before turning gangrenous (tissue death due to lack of blood supply) (29).

The integrity of microvascular endothelium and its component plays a major role as a vascular barrier (i.e., between circulating blood and vessel wall). Therefore, SVD is frequently associated with the endothelium dysfunction that results in arteriolosclerosis and lipohyalinosis. In general, ECs help maintain vascular barrier or health and blood flow (through capillaries and arterioles) in several ways including limiting the platelet or leukocyte aggregation, controlling the vascular permeability from plasma components, and regulating the vascular tone (30). Equally crucial for the ECs to function at their optimum is their interaction or crosstalk with the surrounding cells such as mural cells (i.e., pericytes and vascular SMCs), glial cells (i.e., astrocytes), and immune cells (31).

### Risk Factors and Clinical Relevance of SVD

Previous report had confirmed that hypertension (i.e., systolic blood pressure ≥135 mm Hg), sex (i.e., male), type 2 diabetes mellitus (T2DM), smoking status, and aging (i.e., ≥70 years old) were the main risk factors that can lead to SVD (i.e., in the brain, retina, and heart) (32–34). Another contributing risk factor is the metabolic syndrome including obesity (as of dietary and lifestyle) due to accumulated fat in the abdominal location, hence abdominal obesity. The accumulated fat mediates the synthesis of inflammatory cytokines and causes further inflammation of GIT vasculature (35, 36). Moreover, microvascular complication such as increase of proinflammatory cytokines, vascular endothelial adhesion molecules (VCAMs), and intracellular cell adhesion molecules (ICAMs) has been associated with T2DM (37), hence increasing the risk toward multi-organ SVD.

Apart from that, endothelial dysfunction (in specific, related to cerebral microcirculation) has been associated with the impact of immune system related GIT microbiota, whereby the dietary pattern (i.e., high salt intake) potentially leads to neurovascular dysfunction through GIT initiated T helper cell 17—the cells responsible for tissue inflammation induction and destruction (38). Interestingly, recent evidence suggested that higher SVD incidence is associated with an increased systemic inflammation due to poor sleep quality (39), as well as societal-based depression and loneliness (40–42). Besides, individual(s) with SVD is suggested to suffer from “systemic” condition (27). This is so as SVD is commonly associated with nervous system disturbances such as stroke, cognitive decline, vascular dementia, and gait dysfunction (43–46). However, SVD possesses multiorgan and multidirectional predilection, whereby any organs with similar vascular risks may have the effects. For example, retinal SVD with neurodegeneration-related cognitive decline, retinal microvascular abnormalities associated renal failure, cardiac insufficiency, blindness, lungs, and GIT vascular-based disorders (47–54).

## Cerebral Small Vessel Disease

CSVD is a spectrum of complex and overlapping pathophysiological mechanism of various etiologies affecting the brain microcirculation that can trigger neuronal inflammation and the subsequent neurodegenerative cascade. However, it is generally viewed that CSVD represents pathological consequences of SVD on the brain parenchyma rather than the underlying diseases of the vessels (5). Therefore, the term *cerebral small vessel disease* is generally viewed as the state of brain parenchyma injury (often progressive) that is associated with distal leptomeningeal and intracerebral vessel pathology that resides in poorly collateralized subcortical gray and deep white matter. Moreover, it is mainly due to several focal or diffuse microvasculopathological processes that affect and cause occlusion to the small perforating cerebral capillaries (of sizes 50–400 mm), small arteries (mostly branches of MCAs), arterioles (diameter <0.1 mm), and venules that penetrate and supply the brain cortical and subcortical region (55, 56).

**Figure 2:**
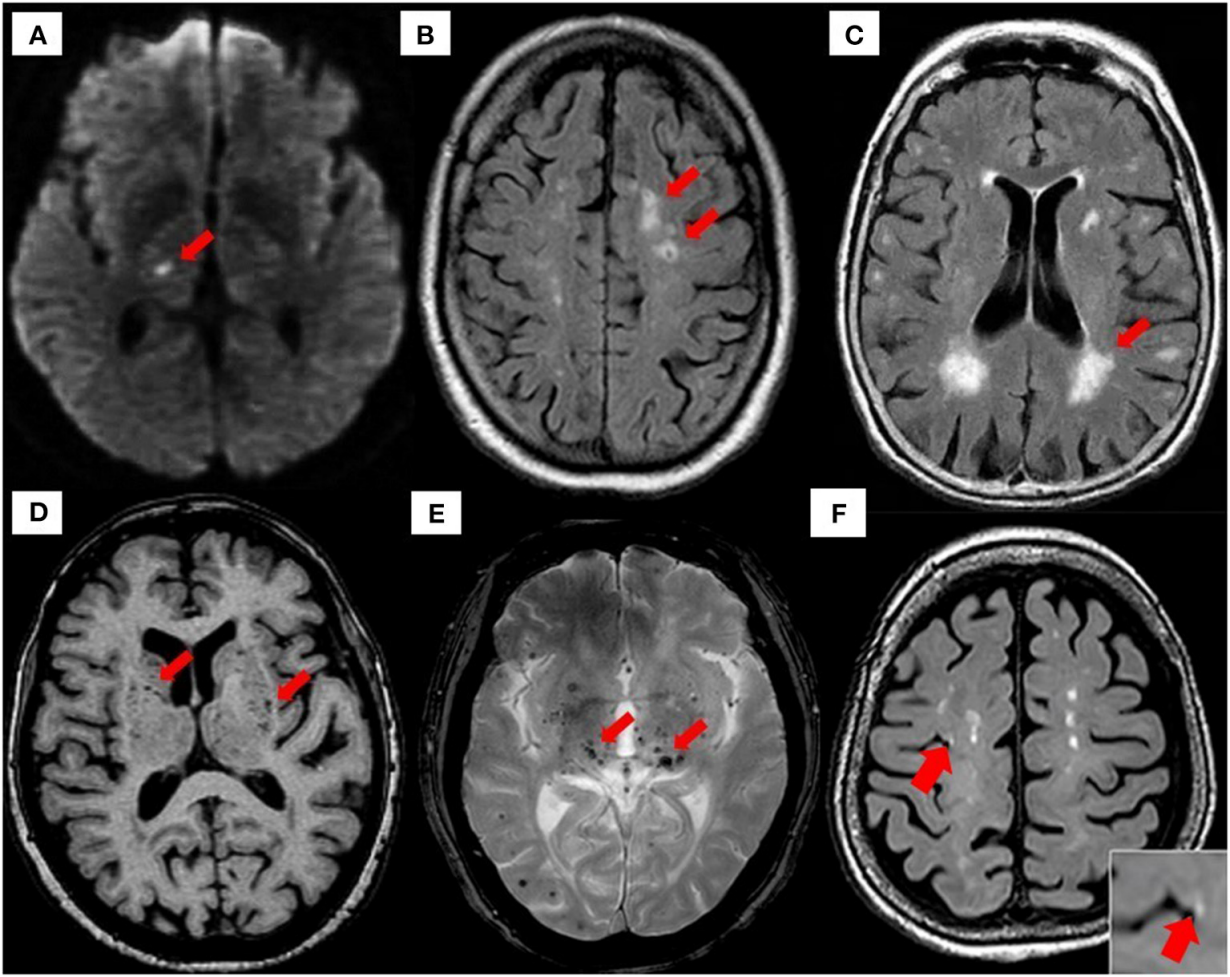
Neuroimaging correlates of CSVD based on STRIVE method. **(A)** Recent small subcortical infarct (RSBI) on diffusion-weighted imaging (DWI) (red arrow). Usual diameter is around 3–15 mm, with hyperintense rim surrounding ovoid cavity. RSBI seen as increased T2-weighted, fluid-attenuated inverse recovery (FLAIR), and DWI signal intensities decreased T1-weighted signal and isointense in T2*-weighted gradient recoiled echo (GRE) signal and susceptibility-weighted imaging (SWI). RSBI is best identified through DWI with usual infarct diameter of ≤20 mm. **(B)** Lacunar infracts on FLAIR (red arrow). Lacunar infarcts appeared as increased hyperintensity in T2-weighted signal, decrease T1-weighted, and FLAIR signal and isointense in DWI. Usual diameter is around 3–15 mm, with hyperintense rim surrounding ovoid cavity. **(C)** White matter hyperintensities (WMHs) of presumed vascular origin on FLAIR (arrow). WMHS seen as increase intensity or hyperintensity on T2-weighted imaging, T2*-weighted GRE and FLAIR (best identified), isointense on DWI, and hypointense (decrease intensity) on T1-weighted imaging. **(D)** Enlarged perivascular spaces (PVS) on T1-weighted imaging (red arrow) with usual diameter of ≤2 mm. PVS is seen as decrease FLAIR and T1-weighted signal intensity, with increase T2-weighted signal. Meanwhile T2*-weighted GRE and DWI appeared isointense, and they also appeared in similar signal intensity with cerebrospinal fluid (CSF). **(E)** Cerebral microbleeds (CMBs) on T2*-GRE (red arrow). CMBs are small, rounded areas of signal void with blooming, whereby they were visualized as isointense T1- and T2-weighted signal, FLAIR, and DWI. They are best identified under T2*-weighted GRE or SWI as reduced signal intensities. Usual diameter is around ≤10 mm (mostly 2–5 mm). **(F)** 3-T MRI representation of cortical microinfarcts (red arrow) on T1-weighted (hypointense) [images A–E, reproduced with permission from Mustapha et al. (57), image F is adapted from Takasugi et al. (61)].

There are several etiopathogenic classifications of CSVD. However, the most well-recognized forms of CSVD are the amyloidal CSVD [e.g., sporadic and hereditary cerebral amyloid angiopathy (CAA)] and non-amyloidal CSVD including age-related and vascular risk-factor–related SVD (i.e., arteriolosclerosis and age) (56). Other less common forms of CSVD include inherited or genetic (monogenic) CSVD that is recognizably different from CAA [i.e., Fabry disease and cerebral autosomal dominant arteriopathy with subcortical ischemic strokes and leukoencephalopathy (CADASIL)], inflammatory and immunologically mediated CSVD, venous collagenosis, and other CSVD (i.e., non-amyloid microvessel degeneration in AD and postradiation angiopathy) (57). Clinical diagnosis of CSVD typically takes the form of acute lacunar infarct and, less commonly, as intraparenchymal hemorrhage, with neuroimaging findings such as white matter hyperintensities (WMHs) of presumed vascular origin, cerebral microbleeds (CMBs), cortical microinfarcts, lacunar infarcts, and recent subcortical brain infarcts (RSBIs) and enlarged perivascular spaces (PVS), or pathological phenomena with multifaceted etiologies (55, 58, 59). However, the lack of standardization and consistency in neuroimaging techniques leads to the development of STandards for Reporting Vascular changes on nEuroimaging (STRIVE), aiding in the imaging-based visual identification and classification of CSVD spectrum (60) (see Figure 2 for neuroimaging correlates of different CSVD manifestation based on STRIVE method).

### Risk Factors of CSVD Manifestation and Their Clinical Relevance

There are several and complex known risk factors toward development and progression of CSVD manifestation. For example, increased imaging loads of WMHs, lacunar infarcts, and RSBI were associated with lifetime exposure toward cardiocerebro(micro)vascular risks such as metabolic syndrome (i.e., hypertension, obesity, hyperlipidemia, dyslipidemia), lifestyle (i.e., smoking, alcohol abuse), and T2DM that posed a higher odd for acute ischemic (lacunar) strokes (62). However, age has served as one of the most significant determinants of the onset, proportion, and progression of all CSVD manifestations [for instance, being prevalent with healthy aging (~6%) in the case of CMBs] (63). Higher risk of CMBs has been found in individuals with symptomatic cerebrovascular disease such as ischemic stroke and intraparenchymal hemorrhage (63). Meanwhile, genetic factors such as *NOTCH3* gene (chromosome 19) mutation as seen in CADASIL; mitochondria DNA mutation as seen in mitochondrial encephalomyopathy, lactic acidosis, and stroke-like syndrome (MELAS); Fabry disease; and familial CAA increase the burden and prevalence of CSVD (64, 65). Hence, optimizing (micro)vascular risk factors for secondary stroke prevention is undoubtedly warranted.

In addition, most of CSVD manifestation has been demonstrated to increase the risk of vascular cognitive impairment and dementia. For example, previous report had shown that elderly person with hypertension who presented with confluent periventricular and hypoperfusion-based deep WMHs, respectively, had impaired executive function, short-term memory loss, and reduced processing speed, although other neurological and medical tests are normal (66). Moreover, elevated WMHs and CMBs were associated with gait disturbance, i.e., reduction in gait velocity, and stride strength (67, 68), higher urinary syndrome, or disturbance including urinary urgency, nocturia, and incontinence (67, 69). A significantly increased risk toward all subtypes of ischemic stroke (70) and neuropsychiatric syndromes (e.g., depression, anxiety, parkinsonism, mood disturbances, reduced processing speed, and sleep disturbance) also had been linked with the presence of WMHs, CMBs, and enlarged PVS (6, 66, 71, 72). Lacunar stroke had been reported as the outcome of small vessel occlusion-mediated lacunar infarcts (73). Moreover, many individuals with CSVD have been reported to have the occurrence of silent brain infarcts, a consequence of a lacunar stroke in a non-vulnerable brain region with unapparent clinical symptoms. Moreover, acute RSBI may cause secondary effects such as remote cortical thinning due to progressive degeneration of connecting white matter tracts (73). Alarmingly, CSVD manifestation can often be occult in nature and produce no clinical symptom (asymptomatic), hence referred to as “silent” brain infarcts.

Taken together, several cardiocerebrovascular risk factors such as T2DM, metabolic syndrome (i.e., hypertension, obesity), aging, and lifestyle (i.e., smoking and unhealthy diet) have been correlated with and increased the risk toward onset and progression of CSVD. Hence, tackling these risk factors may be beneficial in the therapeutic and preventive measures to regulate the onset and progression of CSVD, ideally from early or young age.

### CSVD as a Spectrum of Dynamic Microvascular Pathomechanism

Relatively small vessels/microvessels served an essential role as part of the neurovascular unit or the blood–brain barrier (BBB) in the central nervous system (CNS). To date, various and intensive investigations have been carried out to study the mechanism of interaction between cerebral parenchyma and its surrounding microvasculature (74). However, it is well-accepted that neurovascular unit or BBB owns the prior role in brain health and plasticity (capacity to recover) from insults that may initiate the pathologic cascade toward NDD. Two classical clinicopathologic representations of CSVD have been suggested: arteriolosclerosis or lipohyalinosis (thickening and/or damage the wall of arterioles), and occlusion of cerebral penetrating arteries (75). However, it is now recognized that most of the macrostructural manifestations in CSVD are reflections of the probable underlying of mesostructural responses such as cerebral microcirculation flow obstruction (intrinsic or extrinsic). For instance, the arteriolar occlusion or narrowing resulted in ischemia as seen in small lacunar infarct in the classical CSVD clinical spectrum.

**Figure 3:**
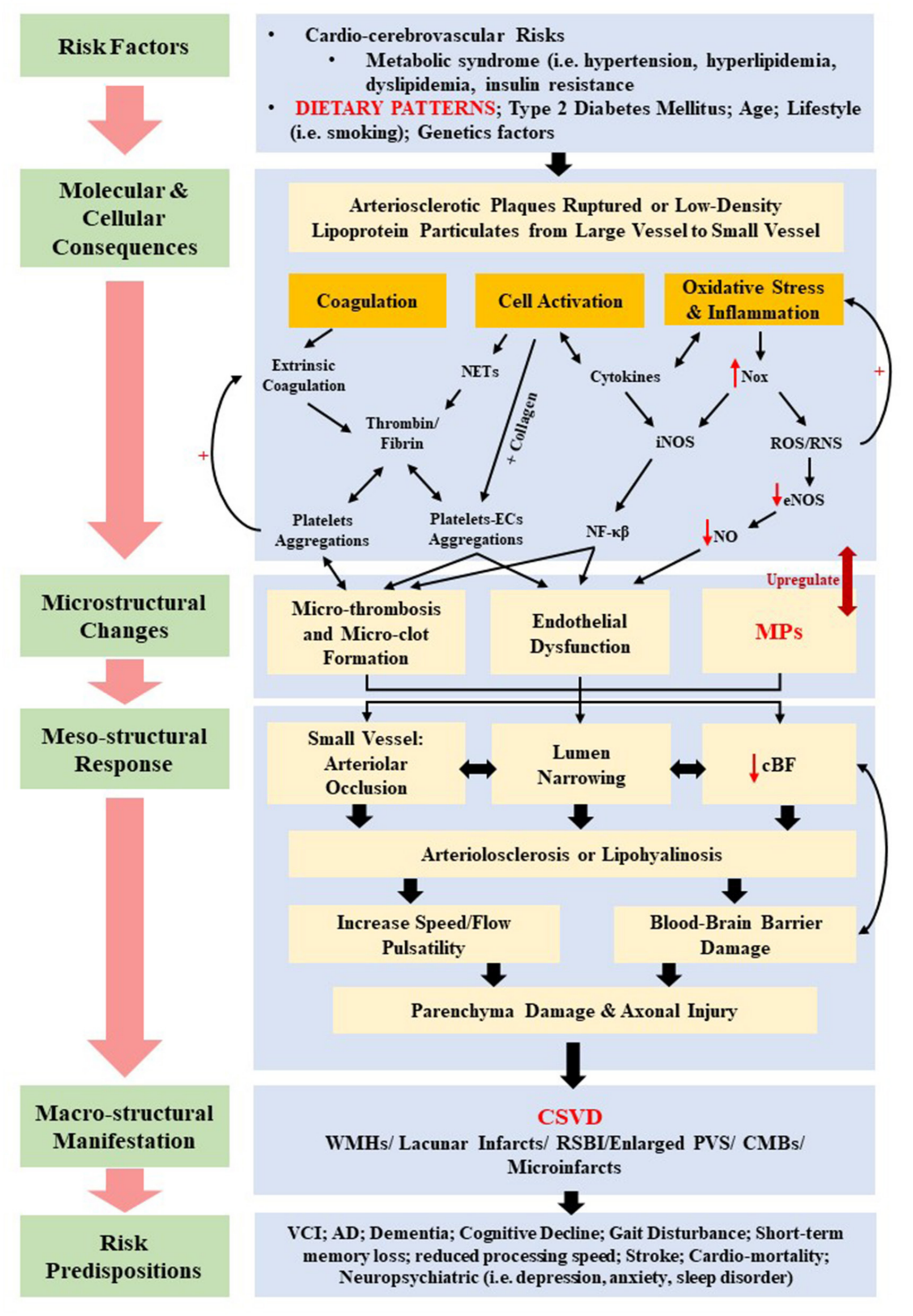
Summary of the proposed overlapping pathomechanisms of cerebral small vessel disease (CSVD) through coagulation, cell activation, endothelial dysfunction, and inflammation. cBF, cerebral blood flow; CMBs, cerebral microbleeds; ECs, endothelial cells; eNOS, uncoupled endothelial nitric oxide; iNOS, inducible nitric oxide synthase; MPs, microparticles; NETs, neutrophil extracellular traps; NF-κβ, nuclear factor κβ; NO, nitric oxide; Nox, nitric oxide synthase oxidase; ROS, reactive oxygen species; RNS, reactive nitrogen species; RSBI, recent subcortical brain infarcts; PVS, periventricular spaces; WMHs, white matter hyperintensities.

Various physiopathologic changes (i.e., the mesostructural responses) of CSVD not only give rise to cerebral parenchyma damage (i.e., axonal injury, neuronal apoptosis, demyelination, and oligodendrocyte damage), but also to neurological symptoms, clinical signs, and multifaceted neuroimaging findings (76). Nonetheless, the underlying pathomechanism of CSVD remains contentious despite the growing insights from histopathological, epidemiological, physiological, and imaging studies. Insights on the current pathomechanism CSVD can be viewed from molecular and cellular consequences of several systemic dysregulations, which include coagulopathy, elevated microthrombosis, genetic mutation, increased cellular activation, inflammation, and oxidative stress, all of which contribute toward the corresponding cerebral microstructural changes such as endothelial dysfunction, altered cBF, and breakdown of BBB. Figure 3 summarizes the current pathomechanism of CSVD through coagulation, cell activation, endothelial dysfunction, and inflammation. Figure 3 also emphasizes on the proposed overlapping and multifaceted risk factors that may contribute to the detrimental macrostructural CSVD manifestations, with a specific highlight on the dietary patterns and MP formation as further elaborated in this review.

#### Coagulation and Microthrombogenesis

In general, the coagulation process or pathway serves to maintain hemostasis or to control bleeding, promote healing, and prevent spontaneous bleed (77). The coagulation pathway is controlled by certain naturally occurring inhibitory elements or anticoagulants such as protein S, protein C, antithrombin, and tissue factor pathway inhibitor (TFPI) that control and limit the formation of clot to prevent propagation of thrombus/microthrombus or further thrombosis/microthrombosis (77). Altered procoagulant properties of such coagulation factors would stir imbalance in the pathway, either with increased or decreased activities of a given factor (78). Generally, the thrombogenic elements of coagulation factors are produced from two sites: the vessel wall [i.e., tissue factor (TF), exposed endothelium, and collagen] and the circulating elements [i.e., platelets, platelet activating factor, prothrombin (factor II), fibrinogen (factor I), von Willebrand factor (vWF), and numerous clotting factors]. Certain events such as physiological disturbance, blood abnormalities, infection, elevated proinflammatory cytokines activities, and disturbance in the primary hemostasis (i.e., platelet plug formation at the insulted site of exposed ECs of the vessel wall) would result in the imbalance of the coagulation system, hence termed as coagulopathy (79, 80).

In microcirculation, whereby the arteriosclerosis and/or arteriolosclerosis is the major culprit in CSVD, the platelets may circulate in resting state. However, upon stimulation (i.e., by ruptured arteriosclerotic plaque or embolism from larger vessel) or activation (even at early stage of disease process), platelets can aggregate by intraplaque components such as TF, collagen, and vWF, or by soluble platelet agonists or vasoactive substances [i.e., thrombin, adenosine diphosphate (ADP), serotonin, or thromboxane A_2_ or B_2_] that promote microthrombogenesis (81). Moreover, platelet activation and aggregation lead to further release of thrombin, hence elevating the activation of coagulation cascade and subsequent synthesis of stable cross-linked fibrin clot or mesh. The formation of fibrin has been shown to increase the coagulation activity whereby the elevated level of alternative marker for thrombin generation such as fibrinopeptide-A has been associated with cerebral infarction (82). Systemic microcirculation coagulation cascade can be activated at early disease process, and platelet activation is the main player in microthrombi formation and its plausible effect on pathogenesis of CSVD.

Small transmembrane glycoprotein or TF facilitates the microthrombosis in microcirculation. In coagulation systems, the extrinsic pathway or the TF pathway is activated once ECs released the TF following damage to the vessel. The TF hence activates thrombogenic element factor VII into factor VIIa that will activate factor X into Xa, resulting in fibrin synthesis. TFPI can interfere and inhibit this pathway. Moreover, TFs are secluded in arteriosclerotic particulates, hence allowing the exposure of TF in microcirculation, leading to formation of microthrombus. Alongside TF, the exposed collagen also facilitates the microthrombosis through glycoprotein (GP-Ia/IIa)–mediated platelets–ECs adhesion, hence activating factor X into Xa leading to microthrombosis and fibrinogenesis (83). Thereby, the balance between prothrombotic factors and endogenous fibrinolysis determines whether the microthrombus progresses into larger thrombus, propagates, or dissolutes (84). Another important component that activates and enhances the contact and prothrombotic pathway, respectively, is the cell-free DNA and histone neutrophil extracellular traps with exposed TFs that present and propagate as part of the intravascular thrombi, hence triggering the generation of thrombin (85, 86). Collectively, platelets and/or neutrophils activation and aggregation could give rise to generation of intra-arterial thrombus or microthrombus and form the basis for arteriomicrothrombotic disease such as CSVD.

In the case of CSVD, activated platelets and microthrombi formation initiate the narrowing of the arterial wall, as well as upregulating the proliferative arterial wall changes (87). Meanwhile platelet aggregation possibly releases the vasoactive substance, resulting in SMC constrictions, hence narrowing the arterial wall (88). Moreover, microthrombi consist of white thrombi of aggregated fibrin, and platelets have been observed to strengthen its association with intraparenchymal small vessel microclot or microthrombosis seen in cerebral ischemia or infarcts (89, 90). Microthrombosis-mediated cerebral microcirculatory dysfunction has been suggested as an outcome of intraparenchymal small vessel dilation to compensate the reduction in perfusion from peripheral pressure of larger arteries. This happened as small vessels trying to optimize the dilation process to maintain the cBF following the arterial lumen narrowing (82).

Moreover, increasing evidences have shown that reduced ability of small vessel to self-regulate cBF (due to aging and the presence of chronic hypertension) is subjected to various systemic blood pressure levels and increased arterial stiffness that would cause an increased speed and flow pulsatility in cerebral arteries and arterioles (91). In addition, the regulation of cBF is also mediated by nitric oxide (NO) signaling, whereby reduced NO is a marker for endothelial dysfunction and altered cBF (92). Thus, these hemodynamic changes may lead to microstructural and mesostructural changes and response, respectively, such as endothelial damage in the BBB and alter its permeability through an increase of the shear stress (93), which will be discussed in the foregoing section. Hence, the BBB breakdown is thought to be another pathogenesis feature of CSVD (93, 94), as hinted in Figure 3.

#### Circulating Cell Activation and Endothelial Dysfunction

As discussed, the cardiocerebrovascular and cardiometabolic risk factors such as T2DM and metabolic syndrome {i.e., dietary patterns, hypertension, abdominal obesity, dyslipidemia [elevated low-density lipoprotein (LDL) and triglycerides and reduced high-density lipoprotein]} had major global impact on development of arteriosclerosis and/or arteriolosclerosis disease, resulting in coronary heart disease and cerebral ischemia (95). Thereby, cellular activation and endothelial dysfunction have been described as the major implication of these risk factors.

It is known for larger vessel circulation that LDL can dissociate into smaller particulates or particles, hence embolizing to smaller vessel microcirculation, which is termed LDL modification (81). Therefore, the infiltration of these smaller particles causes the endothelial dysfunction in large or small vessel. This endothelial dysfunction is followed by EC activations that elevate the subsequent release of proinflammatory cytokines to potentiate host of leukocytes recruitment (i.e., monocyte, T lymphocytes, and macrophages) on the endothelium that further promotes the formation and stability of microthrombus (96). Moreover, monocyte can differentiate into macrophages, which aided in the mechanism of lipid uptake from the circulation. As the endothelial dysfunction ensued, the proinflammatory cytokines may further activate the ECs, hence increasing the expression of adhesion molecules such as VCAM-1, ICAM-1, and even EC-derived MPs (EDMPs) subpopulation such as cluster differentiation 62 (CD62E) or E-selectin. The adhesion process eventually acts on and weakens the ECs and its barriers that line the microvessels lumen. These activated cells distort the functions of EC barriers through the alteration of junctional protein of ECs cytoskeleton or along the width of intercellular junction (81).

Apart from leukocytes, platelet activation also largely contributes to the formation of microthrombus in arteriosclerosis and/or arteriolosclerosis. In response to inflammatory signal, damaged endothelium released the vWF, hence increasing the capacity of platelet activation and binding to vWF. Ensuing platelet activation is the releasing of platelet-derived MPs (PDMPs) CD40, and CD62P (or P-selectin) that bring surface adhesion molecules provoking the platelets and activated platelets by-product aggregation with leukocytes, hence adherence to endothelium promoting microthrombosis and arteriosclerosis (97). Moreover, activated platelets also elevate the synthesis of soluble vasospastic substance such as thromboxane A_2_ or B_2_ and ADP; the synthesis is possible after platelet binding with plasma fibrinogen. These substances elicit the platelets and platelets–monocytes aggregations from inside of arterioles vessel and have been used as markers for onset and progression of arteriosclerosis and/or arteriolosclerosis (82, 98). In addition, the ruptured arteriosclerotic plaques from larger vessel also may embolize and contribute to the instability of the aggregates and microthrombus and upregulate the small vessel systemic inflammation mediated by leukocytes and platelets (99). Aside from cellular activation, endothelial dysfunction can be initiated through the disturbance in the function of microvessel itself as a result of systemic or mechanical stress, leading to microthrombosis. For example, increase in P-selectin and NO in arteriolar endothelium has been associated with microthrombosis (100). Preclinical study had shown that the constriction of arteriolar lumen is due to microthrombosis whereby the intensity of the microthrombosis determined the level of constriction (100). Moreover, the damage in the function of arterioles can lead to local microthrombus formation.

Therefore, circulating cell activation and endothelial dysfunction have long been thought to be the main factors that contribute to the pathogenesis of CSVD. Several studies have shown elevated biomarkers of endothelial dysfunction related to CSVD such as reduced production of NO, resulting in arteriolar constriction (101, 102). Other known manifestations of endothelial dysfunction are hypoperfusion or reduced cBF (103) and increase BBB breakdown or permeability (104) (Figure 3).

#### Oxidative Stress and Inflammation

The risk factors and causes of oxidative stress and arteriosclerosis and/or arteriolosclerosis in the pathomechanism of CSVD are topics with active investigations. In addition, certain health conditions, diet, and lifestyles may contribute to the development and progression of arteriosclerotic and/or arteriolosclerotic CSVD such as dyslipidemia, T2DM, aging, and unhealthy lifestyle (i.e., unhealthy diet, smoking, and sedentary living). Moreover, several studies had shown the association of detrimental effects of oxidative stress [i.e., through nicotinamide adenine dinucleotide phosphate (NADPH) on the endothelium-dependent NO signaling] toward pathogenesis of CSVD (105, 106).

As discussed, the inflammation and oxidative stress may result from increased inflammatory response from the endothelium (i.e., endothelial dysfunction) and cellular activation. Hence, oxidative stress has been associated with the pathogenesis of CSVD as in arteriosclerosis (107). Microthrombus and/or LDL particle aggregates on the small vessel endothelium are susceptible to oxidative and enzymatic modifications by reactive oxygen species (ROS) [i.e., superoxide (O_2_**·**^−^), hydrogen peroxide (H_2_O_2_), and hydroxyl radical (**·**OH)] and proinflammatory cells (95). ROS also induced the imbalance between antioxidants (i.e., EC-derived glutathione peroxidase, catalase, and superoxide dismutase) and pro-oxidants in age-related NDD, whereby the oxidative stress occurs due to NADPH oxidases (Nox)-mediated pro-oxidants overproduction and altered activity of antioxidants enzymes (108). Apart from ROS, the reactive nitrogen species (RNS) also contribute to cerebral vascular oxidative stress, as both ROS and RNS are mainly synthesized by mitochondria activity and certain pathways including NO synthase (NOS) and oxidase enzyme [i.e., NOS oxidase (Nox), uncoupled endothelial NOS (eNOS), cyclooxygenase (COX), lipoxygenase, xanthine oxidase, myeloperoxidase]. However, eNOS is essential in production of endothelium NO, hence also contributing to beneficial or protective role in the regulation of vascular tone, unlike eNOS dysfunction that results in the release of superoxide from ECs (107).

Furthermore, ROS elevate the inflammatory response that influences the progression of clots or thrombus, increase proinflammatory cytokines [i.e., interleukins (IL-6 and IL-8), tumor necrosis factor α (TNF-α), and monocyte chemoattractant protein 1 (MCP-1)] and endothelial function, and increase expression of vascular adhesion molecules (i.e., ICAM-1 and VCAM-1) (109). Subsequently, elevated level of RNS and ROS has been associated with oxidative stress–mediated cell migration and proliferation, DNA damage, necrosis and apoptosis, cellular autophagy, endothelial dysfunction, elevated level of oxidized LDL, and endoplasmic reticulum stress (110). Following overproduction of proinflammatory cytokines and inducible NOS (iNOS) is the activation of transcription factors [i.e., nuclear factor κβ (NF-κβ) and/or nuclear factor (erythroid-derived 2)-like 2 (Nrf2)] and signal transduction cascades (111) that further stimulate the release of cytokines and chemokines, hence increasing inflammation (112). However, NO is able to inhibit the expression of NF-κβ and adhesion molecules; hence, NO serves as crucial anti-inflammatory, antithrombotic, antihypertensive, and antiplatelet aggregation and important for vascular vasolidation (95). Apart from that, NO serves as a modulatory agent for the function of EC barriers whereby, NO modulates the activity of Rho-kinase in cerebral microvasculature and is associated with increase inhibition of NOS (113). Under pathological condition, reduced NO initiates the vicious cycle of reduced NOS to increase the Rho-kinase activation and *vice versa* (114). Hence, maintaining the adequate level of NO is crucial to reduce NO by eNOS to prevent endothelial dysfunction (i.e., elevate the EC monolayers permeability as a response following disruptions of adherent junction and stress fiber formation), whereas overproduction of NO by iNOS leads to an increased expression of proinflammatory factors (115).

Additionally, ROS may act on the ECs inducing the disruption of interendothelial junction, gap formation, actomyosin contraction, and altered phosphorylation or expression of junctional adhesion molecules (115, 116). Furthermore, released cytokines induce inflammation of ECs through extracellular matrix degradation followed by BBB breakdown (104). In addition to the endothelium, there exists cross-talk among cellular components of the BBB, such as pericytes, astrocytes, and oligodendrocyte precursor cells (OPCs) that are implicated in the microvascular damage as precursors for the onset and progression of CSVD (117, 118). In relation to this, reduced white matter integrity due to changes in oligodendrocytes has been shown in CSVD, whereby the ECs–OPCs signaling became compromised and altered the ECs' ability to secrete the releasing factor crucial for the growth and survival of OPCs that eventually caused oligodendrocytes prone to damage (119). An increased BBB damage and permeability further induced the degradation of basement membrane of ECs and accumulation of extracellular matrix components leading to stiffening of vessel wall (120). Moreover, BBB breakdown will intensify with the accompanying increased in the deposition of blood component such as platelets, MPs, and fibrin. Several studies showed that changes in walls of small vessels in the brain due to BBB breakdown lead to ischemic events, classified as WMH, lacunar infarcts, and CMB manifestation of CSVD (7, 93, 94) (Figure 3).

Therefore, the interactions of multiple BBB components are likely to play a crucial role in the discovery and development of new prevention steps and therapies for CSVD. Thus, endothelial dysfunction, BBB breakdown, altered cBF, and impaired cerebral autoregulation due to disturb coagulation system, cellular activation, oxidative stress, inflammation, and microthrombosis are thought to be the major players to the development and progression of CSVD, although another or other potential player(s) is still being sought. One such player is cellular-derived circulating MPs.

## Microparticles—From Peripheral to Central

There has been growing recent interest in the identification and quantification of cellular debris such as MPs as biomarkers for their potential to inform the natural history of development and progression of several diseases including cardiocerebrovascular disease, GIT disease, cancer, metabolic disease, and sepsis. Flow cytometry (FC) is the most widely method to measure MPs and has major advantages over the other techniques in that each MP (and its subpopulations) is quantified individually based on their antigen expressions (121). However, to date, there remains lack of consensus on such standardization between centers, in measuring MPs using FC due to complex and multifaceted nature of MPs. The development of standardized MPs technologies would permit a direct comparison of results between studies and would lead to a greater understanding of MPs in health and diseases.

Besides FC, other MPs assays include single-particle assays and bulk assays (Table 1). Single-particle assays include atomic force microscopy (122) and high sensitivity microscopy (123). These two procedures can be used for an accurate determination of MP size and shape but cannot be used for routine analysis of clinical samples as it can be rather costly to run and maintain (122). In contrast, bulk assays include immunoassays, functional assays, and hybrid assays that detect antigens expressed on MPs (124), PS/TF dependent procoagulant activity (125), and prothrombinase activity (126), respectively. However, bulk assays do not provide size information or single-particle counts (121). Other available MP analysis techniques, although much less popular, include dynamic light scattering (127), high-performance liquid chromatography (128), capillary electrophoresis (129), and mass spectrometry (130). Overall, FC has major advantages over the other techniques in that each MP is interrogated individually and allows for the identifications and quantification of MP subpopulation based on antigen expressions (as summarized in Table 1).

**Table 1:** Profiles of multiple techniques for detection and characterization of MPs.

**Technique**	**Quantification (bulk quantification)**	**Enumeration (single-particle counting)**	**Origin**	**Specificity**	**Sizing**	**Cost/complexity of instrumentation**	**Practicability**
FC	++	+++	+++	++	+	–	++
Immunoassays	+++	–	+	+	–	+	+++
Functional assays	+++	–	+	+	–	+	+++
EM	–	+	+	+++	++	–	–
DLS	++	–	–	–	++	+	+
RICM	–	+	+	+	++	–	–
AFM	–	++	+	++	+++	–	–

*Number of “+” represents the strength of the techniques, whereas “–” represents the weakness of the techniques. AFM, atomic force microscopy; DLS, dynamic light scattering; EM, electron microscopy; FC, flow cytometry; RICM, reflection interference contrast microscopy*.

### MPs—Definition, Formation, and Compositions

**Figure 4:**
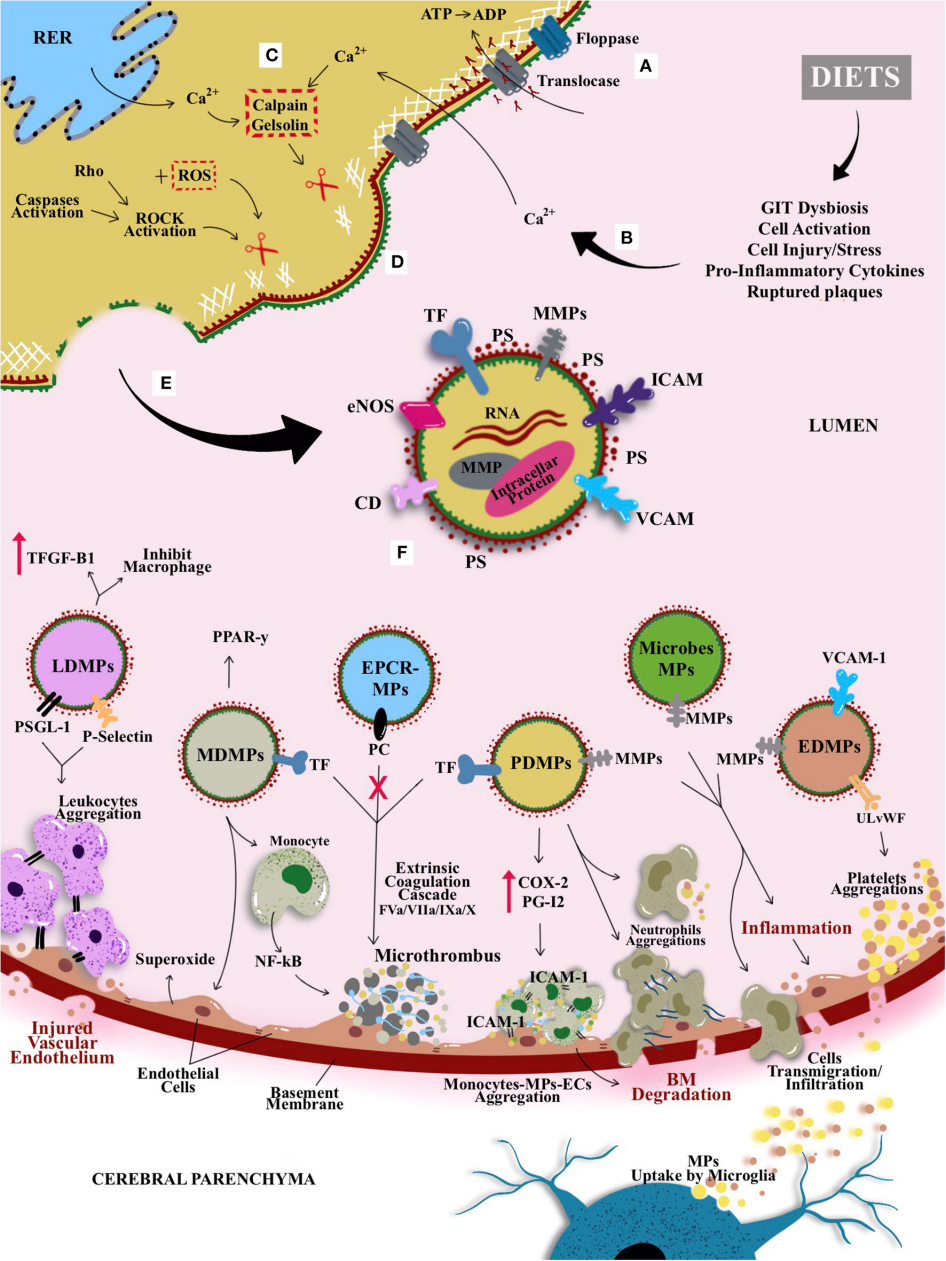
The general mechanism of MP formation, its mode of action, and the proposed possible impacts of diets on MPs that could be linked with CSVD. **(A)** Active translocase transporting phosphatidylserine (PS) from outside to inside layer through adenosine triphosphate (ATP)–dependent manner. **(B)** Modifiable cardiocerebrovascular risk factors (with emphasis on dietary patterns in this review) are known to induce cellular activation or other cellular stressors (e.g., increased cytokines and from peripheral and GIT dysbiosis). **(C)** The activation causes an increase in intracellular cytosolic calcium release by stressed rough endoplasmic reticulum (RER) and acquired from extracellular space. Hence, activates enzymes calpain and gelsolin that cleave cell membrane cytoskeleton. **(D)** The cleaved cytoskeleton causes inactivation of translocase and hence induces phospholipid “flip-flopping.” **(E)** Externalization of PS produces MPs that bring their parent surface molecules and protein antigens. **(F)** MP productions can trigger series of microthrombotic cascades that could be linked to the mechanism postulates on CVSD risk predisposition/prevention that could be modulated by dietary pattern. For example, leukocyte-derived MPs (LDMPs) expressed P-selectin glycoprotein ligand-1 (PSGL-1) and platelet P-selectin on their surfaces and hence aided the aggregation of TF-bearing leukocytes at the site of vascular or microvascular injury. Besides, LDMPs were also able to inhibit macrophages activation and releasing transforming growth factor β1 (TGF-β1). Monocyte-derived MPs (MDMPs) are known to influence the activity of macrophages and monocytes by enhancing the expression of peroxisome proliferator-activated receptor γ (PPAR-γ) protein. However, MDMPs also upregulated superoxide anion production on endothelial cells (ECs) and activation of nuclear factor κβ (NF-κβ) in monocytes that enhance microthrombosis. Most of the MPs, especially platelet-derived MPs (PDMPs) serve as precursor for microthrombus formation by providing catalytic surface for the prothrombinase enzyme complex (i.e., involving factors IXa/Va/VIII/Xa). PDMPs elicit the *de novo* expression and production of inflammatory molecule or agent such as cyclooxygenase (COX-2) and prostacyclin (PG12) that enable the monocytes-MPs-ECs aggregations through intracellular adhesion molecules (ICAM-1) to further elevate the basement membrane (BM) degradation and formation of microclot. Once PDMPs had a close contact with neutrophil, it can bind and increase neutrophil aggregations and elevate neutrophil phagocytic activity. This is followed by an activation of ECs or GIT dysbiosis, as they released endothelial-derived MPs (EDMPs) and bacterial or microbiota-derived MPs that express proteases proteins such as MMP-9 and MMP-2 to enable the invasion toward vasculature through disruption of BM. Disrupted BM enables cellular or molecules transmigration or infiltration; for example, MPs bridging through BBB, may undergo reuptake by microglia from cerebral parenchyma. Alongside proinvasive MMP-9, EDMPs bring ultralarge von Willebrand factor (ULvWF) monomers that upregulate the platelet aggregations to ECs and hence activate the ECs and endothelium dysfunction. Moreover, activated protein C induced the synthesis and release of ECs–protein C receptor (EPCR)–derived MPs that bring functional and actively bound protein C to aid the inhibition of factor Va and factor VIIIa in the common pathway of coagulation cascade leading to thrombogenesis.

MPs represent one of the types and classifications of microvesicles—with an anucleated phospholipid bilayer. Apart from MPs, other classes of microvesicles include exosome and ectosome, which can be distinguished based on their size, composition, and origin. For instance, exosome is considered as the smallest microvesicles with the size ranging from 30 to 100 nm, whereas apoptotic bodies or large membrane blebs range from more than ≤5 μm in diameter (131, 132). However, this review focuses on MPs or ectosomes that are anucleate, small, and membrane-enclosed extracellular particles (133–136). Ranging from 0.1 to 1 μm in diameter, MPs are derived from direct deformation of cell plasma membrane and cell membrane phospholipid exocytic blebs that are released from the cell surface by proteolytic breakdown of the cytoskeleton, triggered by various mechanisms such as cellular activation, oxidative stress, inflammation, injury, or apoptosis. In this context, factors such as different agonists, thrombin, serine proteases, collagen, proinflammatory cytokines, and physiological shear stress, which are known to contribute to cellular activation, would further promote the secretion and aggregation of MPs (135, 137–139). In contrast, during apoptosis, the apoptosis-induced MP release is stimulated by the caspase-mediated Rho effector protein and the Rho-associated coiled-coil containing protein kinase 1 (ROCK 1), as well as by thrombin and TNF-α (140). Figure 4 illustrates the general mechanism of MP formation and its mode of action, while it also introduces the proposed possible impacts of diets on MPs that could be linked with CSVD (as previously hinted in Figure 3). A converging proposed plausible link between diets, circulating MPs and CSVD manifestation is further delineated in *Diets and Circulating MPs—Proposing the Link With CSVD*.

MPs are heterogeneous and can be produced from multiple sources (or parental cells) within blood circulation, i.e., from platelets, erythrocytes (or RBCs), leukocytes (white blood cells), monocytes, ECs, and SMCs (141). Also, MPs can be present in various body fluids such as saliva, urine, bile, cerebrospinal fluid, and synovial fluid (142). MPs are identified by the presence of cell surface marker phosphatidylserine positive (PS^+^), although PS negative (PS^−^) is recently recognized (143). Moreover, in the blood circulation of healthy individuals, MPs are present in low level, with 70–90% of MPs represented by PDMPs (144). MPs are composed mainly of cytosol and enclosed by globose phospholipids bilayer, whereby their cytosol may include RNAs [i.e., non-coding small interfering ribonucleic acid, messenger RNA (mRNA), and micro-RNA (miRNAs)] (145, 146), enzymes, and cytoskeletal proteins of their parental cells, but are anucleate and lack synthetic capacity. However, to date, there is no evidence of DNA presence in MPs luminal space, although a trace of DNA had been found in exosomes and apoptotic bodies (147).

Given that MPs carry their own parental membrane proteins or markers, these are used to identify their cell of origin or subpopulations. For examples, cluster differentiation 41 (CD41) is to identify PDMPs, CD235/CD235a for RBCsderived MPs (RDMPs), CD31/CD146 for EDMPs, and CD45 for leukocyte-derived MPs (LDMPs) (148). Interestingly, PDMPs bring more than 40 membrane integral protein or glycoprotein characteristic of platelets, such as integrin β1 (CD29), αIIbβ3 (CD41), and P-selectin (CD62P). PDMPs and EDMPs also bring proinvasive or proinflammatory matrix metalloproteinase proteins (MMPs-2/9). Most of these proteins serve as adhesion molecules that stimulate the EVs internalization by these cells (144). Meanwhile, RDMPs are the smallest (~0.15 μm) compared to other cell-derived MPs, whereby their surface consists of residual hemoglobin (20% from parent RBCs) (149, 150) (see Table 2 for details).

**Table 2:** Microparticles (MPs) subpopulation and their surface markers.

**Parental cells**	**MPs**	**Surface markers/cluster differentiation (CD)**
Platelets	Platelet-derived microparticles (PDMPs)	• CD62P or P-selectins (maker for platelet activation) • CD154 04 CD40L (maker for platelet activation) • CD42b (glycoprotein Ib) • CD42a (glycoprotein IX) • CD41/CD41a and CD63 • CD29 (integrin β1)
Endothelial cells (ECs)	Endothelial-derived microparticles (EDMPs)	• CD31/CD146/CD144/CD105 (maker for apoptotic-derived EDMPs) • CD54/CD106 (markers for EC activation) • CD62E or E-selectins and CD106 (marker for cellular inflammation) • EDMPs markers also expressed on other cell types, such as CD146 (expressed on pericytes and tumor cells), CD54 (expressed on leukocytes), CD105 (expressed on activated monocytes), and CD31 (expressed on activated platelets)
Leukocytes	Leukocytes-derived microparticles (LDMPs)	• CD45 (mostly all LDMPs) • CD14 (monocytes derived, MDMPs) • CD4 (lymphocytes) • CD15 (granulocytes)
Erythrocytes [red blood cell (RBC)]	Red blood cell–derived microparticles (RDMPs)	• CD47 • CD235/CD235a

*CD, cluster differentiation; ECs, endothelial cells; MPs, microparticles*.

In addition, previous studies reported that MPs consisted of identical lipid composition as plasma membrane. However, MPs may have augmented cholesterol or specific enrichment, sphingomyelin, or ceramide, which implies that MPs can be produced or shed from certain region of cellular plasma membrane, cell of origin, and/or pathophysiological properties (149). As aforementioned, majority (if not all) of MPs expose PS^+^ at their outer membrane surface; hence, PS has been used as standard marker of MPs identification (149).

### Notable Roles of MPs in Health

Recent evidence has shown that MPs extend some protective effects in health as part of maintaining the hemostasis. Hence, several subpopulations of MPs could also potentially play a role in mitigating the inflammatory effects. For example, EDMPs contain anticoagulant properties at their surface, which is important to bring balance in hemostasis by counterbalance the thrombosis driven by procoagulant MPs (151). Besides, an *in vitro* study has shown that EDMPs are crucial for maintaining the integrity of vascular wall through the activation of vascular repair (134).

Moreover, in coagulation system (i.e., in common pathway), the activated protein C is able to induce the synthesis and release of EC–protein C receptor–derived MPs, whereby these MPs bring functional and actively bound protein C aiding in the inhibition of factor Va/VIIIa in the common pathway of coagulation cascade (152). Apart from that, certain subpopulations of MPs also possess anti-inflammatory properties; for example, monocyte-derived MPs (MDMPs) are known to influence the activity of macrophages and monocytes by enhancing the expression of peroxisome proliferator-activated receptor γ (PPAR-γ) protein (153). Furthermore, LDMPs also have been shown to possess an anti-inflammatory property, whereby they potentially aided in the downregulation of proinflammatory mechanism in coagulation cascade at an early stage of inflammation (154). Besides, LDMPs are also able to inhibit macrophages activation through the activation of anti-inflammatory macrophage response, i.e., the inhibition of cytokines (such as IL-8), inhibition of TNF-α, and releasing transforming growth factor β1 (154). Interestingly, low level of EDMPs was also found to correlate with thrombin and anticoagulant markers in healthy individuals, raising EDMPs' role in the inhibition of thrombosis (155).

### MP Roles in Coagulation and Microthrombosis-Linking CSVD Correlates

Much of the MPs procoagulant and prothrombotic properties are due to their ability to bind to sub-endothelial matrix (and its components), adhesion with soluble and non-mobile fibrinogen, and coaggregation with platelet aided by a complex and dependent process involving GP-IIb/IIIa (156). As mentioned, PS presence on MPs surface acts as coagulation factors for assembly and binding agent or proteins in coagulation cascade that may lead to a prothrombotic state (137). PS binds to hematopoietic-derived clotting factors through electrostatic interactions between phosphate groups in phospholipids and Ca^2+^ in γ-carboxyglutamic (GLA) domain of clotting factors (157). Factors VII, IX, X, and prothrombin are the clotting factors that contain GLA domain. Therefore, the recruitment of PS bearing MPs and clotting factors aided the aggregation of platelet and synthesis of fibrin and hence for the formation of microthrombus (158). Furthermore, *in vitro* study had shown that combined PDMPs and EDMPs at low levels can also induce the generation of microthrombus (159). Of note, compared to activated platelets (parent cells), PDMP surfaces possessed up to 100 times higher procoagulant properties and higher affinity binding sites for activated coagulation cascade (160, 161). Hence, PDMPs would serve as a precursor for microthrombus formation by providing catalytic surface for the prothrombinase enzyme complex (i.e., involving factors IXa, Va, VIII, and Xa) (158).

Moreover, MPs also bring surface TF, where, for example, MDMPs have been reported to bring active TFs that potentially elevated the extrinsic pathway involving factors VII, VIIa, IX, and X in coagulation cascade (162, 163). In addition, LDMPs expressed P-selectin glycoprotein ligand 1 and platelet P-selectin on their surfaces that lead to the aggregation of TF-bearing leukocytes at the site of vascular or microvascular injury (164). In addition, the formation of EDMPs has also been associated with elevated level of endothelial dysfunction marker such as plasminogen activator inhibitor 1 (PAI-1) and elevated the procoagulant activity and prothrombotic state. This is so because EDMPs contain the expression of ULvWF multimer that enabled EDMPs to induce strong platelet aggregations (165). Therefore, it is plausible to deduce that TF-bearing MPs play an important part in macrothrombus and microthrombus formation. In fact, a study had shown that tumor cell–derived MPs bearing both PS^+^ and TF can be utilized as a biomarker for risk of venous thrombosis in cancer patients (139) (Figure 4).

Thus, in relation to CSVD clinical manifestations, numerous reports linking MP subpopulations as CSVD correlates may well reflect the fact that PS-bearing MPs and clotting factors aided the aggregation of platelet and synthesis of fibrin, which lead to the plausible microthrombus involvement in CSVD pathomechanism (see Table 3 for MPs and CSVD correlates).

**Table 3:** Microparticles (MPs) subpopulation, their surface markers and CSVD correlates.

**Microparticles (MPs)**	**Changes in MP level**	**CSVD correlates**
Platelet-derived microparticles (PDMPs)	• Increase CD42^+^, CD61^+^, CD62P^+^, and CD42a	• 110 patients (mean age, 71.1 ± 7.9 years) with acute-phase cerebral infarction, 34 with small vessel occlusion (166)
	Increase CD41^+^ and CD41^+^/A^+^	• Cerebral infarction attributable to vasospasm in 20 elderly subjects (mean age, 52.2 years), suggesting the consequences of microthrombosis (167) • 40 middle-age subjects (mean age, 44.4 ± 12.2 years) with metabolic syndromes (168)
	• Increase level of CD40L and soluble P-selectin	• Silent brain infarct in subcortical white matter in 15 male healthy obese subjects and 50 male obstructive sleep apnea subjects (more prevalent) (169)
	• Increase CD41^+^	• Middle cerebral artery occlusion in a rat model with cerebral infarction (170)
	• Increase total PDMPs	• In individuals with micro-embolic cerebral ischemia and associated with recent cerebrovascular events as seen in DWI (171)
Leukocytes-derived microparticles (LDMPs)	• Increase CD14	• Related to higher WMHs and the progression of brain atrophy in individuals (*n* = 534, 4 years' follow-up) with vascular disease manifestation (172)
	• Increase CD45^+^ and CD45^+^/A^+^	• An increased risk of arteriothrombotic stroke with individuals with obstructive sleep apnea (173, 174) • Cerebral infarction attributable to vasospasm in 20 elderly subjects (mean age, 52.2 years), suggesting the consequences of microthrombosis (167).
	• Increase CD4^+^/TF^+^	• In individuals with cardiometabolic risk factors such as T2DM and dyslipidemia (175)
	• Increase CD45^+^, CD14^+^, CD4^+^ and CD15^+^	• 76 elderly individuals with ischemic cerebrovascular diseases (176)
Endothelial-derived microparticles (EDMPs)	• Increase CD105^+^/PS^+^, CD54^+^, and CD144^+^	• 41 elderly individuals with mild, moderate to severe ischemic stroke (177)
	• Increase level of CD144^+^, CD31^+^ and CD62E	• 129 elderly individuals [68 with acute ischemic stroke (mean age, 63.59 ± 13.33)] (178)
	• Elevated CD31^+^/A^+^ and lower CD62E^+^	• 101 middle-age individuals with metabolic syndrome (with and without chronic heart failure), suggesting the relevance to neurohumoral and inflammatory activation (133)
	• Increase EDMP bearing VCAM-1 and soluble P-selectin	• 18 individuals with subcortical and periventricular subcortical lesion (179)
Red Blood Cells-derived microparticles (RDMPs)	• Increase CD235^+^ and CD235^+^/A^+^	• Cerebral infarction attributable to vasospasm in 20 elderly subjects (mean age, 52.2 years), suggesting the consequences of microthrombosis (167)
	• Increase CD47* * Least data on its association with CSVD, compared to other MP subpopulations above	• Induced cerebral neuronal cell death *in vitro* (180)

*CD, cluster differentiation; CSVD, cerebral small vessel disease; DWI, diffusion-weighted imaging; ECs, endothelial cells; MPs, microparticles; T2DM, type 2 diabetes mellitus; WMHs, white matter hyperintensities*.

### MPs and Inflammation

The release of MPs into the circulation that ensued tissue or cell inflammation can further aggravate the inflammatory activity (181). MPs can affect microcirculation by potentiating the production and expression of proinflammatory cytokines, chemokines, and ICAM-1 (182) (Figure 4). *In vitro* study had shown that ECs and monocytes' interaction with PDMPs able to elicit the *de novo* expression and production of inflammatory molecule or agent such as COX-2 and prostacyclin (PG12), respectively (183). Another *in vitro* study had shown that EDMPs upregulated E-selectin, ICAM-1, and VCAM-1 and induced the expression and release of proinflammatory cytokines (i.e., IL-6 and IL-8) (184).

Furthermore, within the CNS, microglia are the innate immune cells with diverse roles and functions at their quiescent surveillance, as well as activated states (185–187). However, the traditional classification of M1-proinflammatory/M2–anti-inflammatory microglial phenotypes has been challenged with the emerging evidence, indicating a wide spectrum of microglial activation (188, 189). Microglial function and dysfunction have been indicated in aging and NDD such as AD (188, 190), PD (191), and stroke (192). Three types of microglia and CNS macrophages located around cerebral small vessels have been identified: (i) parenchymal microglia (distal to small vessels); (ii) vessel-associated microglia, which are parenchymal microglia proximal to cerebral vessels; and (iii) perivascular macrophages, which are located in perivascular spaces (193). Microglial activation was found to be associated with BBB leakages and cognitive impairment in angiotensin II–induced hypertensive mouse model (194), and subsequent study showed that inhibition of microglial activation reversed short-term memory impairment in mice (195). Distinct populations of extracellular vesicles have been identified in activated BV2 microglial cells in response to lipopolysaccharide challenge (196). Activated microglia release MPs carrying IL-1β, and these microglia-derived MPs enhanced inflammatory response by transferring inflammatory stimuli to other microglia (197–199). A study by Schindler et al. (200) using cultured human mononuclear phagocytes demonstrated that microglia-derived MPs induced NF-κB activation, leading to the release of proinflammatory cytokines (200). The role of microglia-derived MPs was further substantiated in a study investigating neuroinflammation following brain traumatic injury whereby the MPs (identified through P2Y12/CD45^+^) derived from neuroinflammation that developed in the brain were released into the circulation and initiated neuroinflammation in naive control animals (201). Collectively, these findings highlighted the role of MPs and microglia-mediated neuroinflammation in the CNS.

### MPs and Cell Signaling

Alongside with procoagulant and proinflammatory abilities of MPs, they can also serve as mediators for cell-to-cell interactions and signal delivery between cells. As MPs bring along specific parental membrane receptors, cytosolic proteins, and RNAs, they can stimulate certain target cells to transform and communicate with microcirculation in a way programmed by these contents of MPs (202). For example, PDMPs can stimulate B cells to synthesize specific antibodies such as immunoglobulin G (IgG) by delivering CD154 IgG (203). In addition, PDMPs assisted in monocytes to EC interaction through ICAM-1 that could elevate chemotaxis of monocytoid cells (204). Furthermore, a previous study showed that once PDMPs had a close contact with neutrophil, they can bind and increase neutrophil aggregations and promote neutrophil phagocytic activity (205). Likewise, MPs can be phagocytosed by certain cancer cells (i.e., in lung cancer), hence stimulating the cell to further proliferate, inducing the expression of mRNA for the proinvasive MMP-9, and upregulating the adhesion to ECs, which activated the EC and endothelium dysfunction (206). Following the activation of ECs, they released EDMPs that express proteases proteins such as MMP-9 and MMP-2, leading to vessel invasion through the disruption of basement membrane (207) (Figure 4).

### The Roles of MPs in GIT–Brain Axis

As discussed, although most of the organs are anatomically distinct, they shared a common systemic circulation and blood supply mainly from abdominal aorta, which relates to the brain–heart–GIT axis. This is particularly the case given the emerging debates on the contribution of MPs through GIT-microbiota–derived MPs for GIT immune system and the connection with the heart and the brain.

Certain insult in GIT microbiota (i.e., through substance abuse or infection) has been associated with disturbed immune response and eventually GIT dysbiosis that preceded with metabolic and inflammatory disease (208, 209). Several studies suggest the involvement of systemic GIT-microbiota–derived MPs for these changes. For instance, Shen and colleagues had shown the association of *Bacteroides fragilis*–derived MPs with GIT disease (210), whereas Kang and colleagues linked saccharibacteria or TM7 (i.e., *Akkermansia muciniphila*) bacteria-derived MPs with progression of colitis (211). Therefore, it is plausible to deduce that microbiota-derived MPs may serve as the link to connect between these major organs, i.e., the brain–heart–GIT axis. Similarly, it is plausible that MPs derived from peripheral circulation would assume similar systemic circulation route to reach microcirculation network and hence contribute to the pathogenesis of SVD and NDD including CSVD.

The association or crosstalk between the system in peripheral organ, i.e., GIT microbiota and the brain, is of active research interests (212). Several studies had also described that circulating cells and/or microbiota-derived MPs generated from the peripheral system that enter the systemic circulation and assisted in crosstalk between the cerebral BBB and inflammatory pathways as a trigger for CNS insults (201, 213–215). However, despite the recognized role of peripheral MPs in pathomechanism of CNS disease, the detailed mechanism of MPs breaching the BBB remains elusive, with some insights involving proinvasive or proinflammatory MMP release, reorganization of extracellular matrix, recruitment of inflammatory cells, and regulation of epithelial barrier (216).

In addition, the interaction between the brain and the periphery is a bidirectional communication. This is supported by the evidence from the detection and enumeration of brain-derived MPs in the blood that are likely to have reached cerebral microcirculation and breached into cerebral parenchyma following uptakes by microglial cells (217, 218). For example, GIT or microbiota-derived MPs may bring proinflammatory and degradative enzymes such as MMPs, whereby this molecule enables MPs to be transmigrated into epithelial layer, be circulated in systemic circulation, and reach multiple organs including the brain. Moreover, the disrupted BBB and GIT epithelial layer enhance the inflammatory cargo deposition and cell signaling by MPs (Figure 4). This evidence lends support on the role of MP-mediated transport or breach through BBB as a putative insight on MP-mediated GIT-directed NDD such as CVSD.

### MPs and Related Clinical Syndrome

It is well-accepted that the elevated level of MPs in blood circulation is reflective of their multifaceted roles; for example, higher level of MPs was found in hypertensive patients (219), abdominal obesity (220), myocardial infarction (221), tumor progression and metastasis (222), atherosclerosis (223), and cardiopulmonary bypass patients (160). Previous *in vitro* study had shown that elevated T lymphocytes–derived MPs induced arterial endothelial dysfunction (i.e., reduce expression of NOS) in immunocompromised states (224, 225). Moreover, another studies had shown that MPs can contribute to acute lung injury (226) and inflammatory airway disease (227); in this case, elevated level of MDMPs was enumerated to associate with upregulated proinflammatory IL-8, ICAM-1, MCP-1, superoxide anion production, and activation of NF-κβ in monocytes (153, 227). Interestingly, elevated EDMPs also had been correlated with the severity of endothelial dysfunctions in heart disease, i.e., coronary artery disease and acute coronary syndromes with worst clinical outcomes (133, 228, 229).

In the case of brain disease, MPs have been shown to contribute to both proinflammatory and anti-inflammatory responses in inflammation-mediated NDD including PD, AD, amyotrophic lateral sclerosis, and dementia (230), whereby CNS-derived MPs have been shown to circulate in peripheral circulation and hence may play a role in cerebral immune status by transferring peripheral proinflammatory molecules to CNS (218, 231, 232). Recent evidence also suggested that MP-mediated release of proinflammatory cytokines, miRNAs, and microbial by-products is associated with the onset, progression, and resolution of inflammation-based cerebral injury and NDD (233–235). Therefore, these associations make circulating MPs as pertinent and potential biomarkers of numerous disease onset and/or progression with CNS diseases (228, 236), in particular with microcirculation involvement as observed in CSVD manifestations.

## Diet as Risk Factors for Microthrombosis and SVD

It is well-acknowledged that healthy diet is crucial, and for it to be appealing, such a diet must be nutritious, pleasing, and indulging. As all foods contain variable degree of nutrients or additives, these food elements may be beneficial or detrimental (i.e., increase risk toward chronic disease) to our health. For the past decades, research had focused on a single nutrient consumption by the individual, i.e., protein, fat, carbohydrates, fiber, and sugar. However, as humans, we do not consume a single nutrient as such, but take food as whole. Moreover, nutrients also are associated with one another; hence, focusing on the effect of a single nutrient in food is rather incomplete. Thus, to date, growing research is now focusing on multinutrient interplay in foods and their effects on health, termed as *dietary patterns*. Dietary pattern has been described as the overall diet, type/groups of food and the nutrients therein, the combination/variety, and the quantity/frequency with which the food are habitually consumed (237, 238).

In addition, diet plays an important role in maintaining the homeostasis and hemostasis systems, whereby healthy dietary pattern has been classified as diet with lower concentrations of plasma proinflammatory markers (8). Certain modifications in the dietary pattern could potentially lead to alterations in these systems, notably in individuals who consume less or non-nutritious or unbalanced diets, often linked to the typical Western-type diet, i.e., meat-based with elevated level of proinflammatory markers (9, 10). Modern lifestyles (with physical inactivity and smoking) and unhealthy dietary patterns are recognized modifiable risk factors for metabolic disease, coronary heart disease, and stroke (11) and likely to trigger systemic peripheral events that can influence the development (from early age) to progression (in middle age and elderly) of NDD such as CSVD. A recent systematic review has also linked unhealthy diets with neuropsychiatric disorder such as mental illness (239).

In the current globalization era, metabolic syndrome (syndrome X) (i.e., abdominal obesity, hypertension, insulin resistance, dyslipidemia, and hyperlipidemia) has become a major global health burden as a new non-communicable disease and a risk factor for cardiocerebrovascular disease. This scenario continues to coexist with the social standard of living and influences dietary pattern as a consequence from this social pressure (240). Hence, the foregoing paragraphs will discuss on the range of dietary patterns to date, with their likely effects on the onset and progression of non-communicable diseases such as CSVD.

### Western Pattern Diet

The Western pattern diet (WPD) or modern dietary pattern is classified as a high intake of processed food [i.e., processed meat, red meats, prepackaged foods, and sugary desserts (candy and sweets), refined grains or carbohydrates, fried foods], conventionally raised animal products, eggs, corn (i.e., high-fructose corn syrup), potatoes, high-fat dairy products, and high-sugar drinks. All in all, these consumptions are classified as high intake of saturated and omega-6 fatty acids (SFAs) (241). Moreover, WPD is accompanied by no or low intake of omega-3 FA such as vegetables, fruits, whole grains, nut, grass-fed animal products, fish, and seeds (242). Components in WPD diet tend to be proinflammatory in nature, causing GIT dysbiosis (i.e., alteration in the diversity of GIT microbiota and reduced total bacterial load) and disrupting epithelial barrier structure and function in the GIT system (243).

Additionally, WPD has been widely associated with metabolic syndrome, arteriosclerosis and/or arteriolosclerosis, and T2DM (81, 244). Gross and colleagues reported that refined carbohydrate (i.e., in corn syrup) is associated with T2DM (245). Recent meta-analysis also concluded that higher intakes of food with refined or high-glycemic carbohydrates (seen as high-glycemic index, GI) increased the harmful effects toward T2DM (246). The risk of myocardial infarction also increases with high GI and high SFA by 33% (247). Moreover, highly refined carbohydrate with reduced fiber content found in corn starch, white rice, and white wheat flour has been associated with 55% higher prevalence of T2DM in East Asian population (248, 249). A higher incidence of hypertension and metabolic syndrome has been reported among Asian Indians with higher intakes of refined grain and increased waist circumference (250).

Furthermore, a higher intake of SFA has been associated with an increased endogenous thrombin related to metabolic syndrome (251). Alongside thrombin is the increment of vitamin K–dependent factors (i.e., factors II, VII, IX, and X) and extrinsic TF pathway in coagulation cascade with reduced TFPI, which facilitated microthrombosis formation. Apart from that, high intakes of red meat that is rich with heme iron also increased oxidative stress, epithelial proliferation, and iron-induced hypoxia signaling. Heme iron is known to increase the formation of harmful endogenous *N*-nitroso compound and heterocyclic amine content in GIT (252). Therefore, high intake of processed or unprocessed red meat is associated with higher incidence of vascular microthromboembolism, hence a higher burden of T2DM, risk of metabolic syndrome, colorectal cancer, and stroke (with an increased risk of ischemic stroke by 24%) (253, 254).

### High-Fat Diets/Low-Carbohydrate Diets

High-fat (HFD) or low-carbohydrate diet (LCD) or ketogenic diet is a diet that is rich in fat contents such as SFA (i.e., myristic and palmitic acids) found in animal or tropical oils. HFD also included the low polyunsaturated FA (PUFA) such as linoleic acids (LAs) and α-linoleic acids (ALAs) and monounsaturated FA (MUFA) such as oleic acids (255). Dietary ALA and LA synthesized arachidonic acids (AAs) and docosahexaenoic acids (DHA) in the liver and brain (<1%) (256). The association between the high SFA intake and development and progression of vascular disease is complex because of modulatory effects of fat in both prevention and progression of vascular disease (81). However, habitual HFD individuals had been found to have increased WMH load (i.e., CSVD manifestation) (257). Furthermore, SFA triggers microglial activation to release proinflammatory stimuli by interacting with toll-like receptor 4 (TLR-4) (258). Activated microglia release MPs (197–199), and these microglia-derived MPs have been implicated to exert negative impact in cognition and synaptic plasticity in HFD mice (259).

In contrast, multiple studies had shown the beneficial effects of diets enriched with PUFA and/or MUFA (260, 261). In unesterified forms, AA and DHA cross the BBB through passive transports, and upon entering the brain, they regulate the neuroreceptor-coupled signaling and transcription that serve in modulating the cerebral immunity as they are the mediators for bioactive lipid (262, 263). Sun and colleagues had reported that DHA is beneficial in stroke protection, therapy, and prevention (264). This is due to fact that DHA aided in reducing the neuronal and white matter loss, reducing proinflammatory cytokines, MMP expression, and BBB damage, and regulating the activation of microglial (264). Moreover, DHA reduced platelet aggregation and lag time in healthy individuals (265), hence reducing the risk of microthrombosis. High-MUFA (i.e., oleic acids) diets helped to reduce thrombogenic factor (i.e., factor VIIa and factor VIIc) (266), whereas increased HFD (i.e., higher SFA intake) has been associated with an elevated level of proteobacteria species such as *Bilophila wadsworthia* (GIT dysbiosis), unlike high MUFA that reduced total bacteria in fecal content (267, 268).

Therefore, the interactions between dietary lipid (fats) with microbiota are crucial in the regulation of metabolic changes and systemic and peripheral inflammation. Previous studies proved that the inflammatory pathway from GIT to the brain occurred following the changes in the GIT microbiota (269). This is made possible because SFA (i.e., palmitic acids) can activate the inflammatory response after desensitization of the GIT vagus nerve as seen in microglia-activated TLR4 in hypothalamus (270). In addition, *in vivo* and *in vitro* studies have shown that elevated expression of apoptotic genes and proinflammatory markers (i.e., TNF-α and ILs) with a reduction in brain-derived neurotrophic factor are associated with HFD (i.e., high SFA) (271, 272). Furthermore, Takechi and colleagues reported that BBB damage following high-SFA diets is attributable to elevated neuroinflammation after cerebral microvasculature leakage of peripheral proteins (273).

As mentioned, HFD implies low carbohydrate intakes and that LCD with high protein diets in mice model decreased the amount and function of circulating endothelial progenitor cells (EPCs) (274). However, if LCD (i.e., high unsaturated FA, low in fiber, vitamins, minerals, and polyphenols) is implemented with high PUFA and MUFA, this combination may turn out beneficial and cardioprotective instead. A previous study reported the reduced level of EDMPs (E-selectin), thrombomodulin, C-reactive protein (CRP), and PDMP (P-selectin) in individuals who practiced LCD (275), i.e., likely to reduce the risk toward T2DM and metabolic syndrome, two major risk factors for CSVD.

### Mediterranean Diet

Mediterranean diet (MeDiet) is the type of diet that is characterized by the intake of high portion of vegetables, fruits, nuts, legumes (i.e., peas, lentils, beans, chickpeas, peanuts, and soybeans), olive oils, whole grains, and aromatic spices and moderate to high intake of marine origins (i.e., fish) and low intake of meat and sweetened products (276). MeDiet has been suggested as one of the healthiest and closest model diets toward a healthy diet (11, 277). It is associated with better control of cardiocerebrovascular risk factors such as hypertension (improve blood pressure), glucose metabolism, arrhythmic risk, metabolic syndrome (i.e., dyslipidemia), and GIT microbiota (278). The protective effects of MeDiet are attributable to its high level of PUFAs (from marine origins and plants), MUFAs, minerals, polyphenols [a dietary antioxidants from plants origins and beverages (i.e., green and black tea, coffee, and red wine)], and fiber, while low in SFA and sugar. All these components in MeDiet are associated with anti-inflammatory effects and reduced prevalence of vascular diseases (279), with underlying effects on modulating proatherogenic or arteriogenic and proinflammatory gene expression such as COX-2, MCP-1, and LDL receptor-related protein (LRP1) (280); lowering plasma level of prothrombotic coagulation and inflammation molecules such as ILs (i.e., IL-10, IL13, IL-18) and MMPs (i.e., MMP-9); and decreasing the NF-κβ activation in leukocytes (281, 282).

Marine origins such as fish in MeDiet is the major source of protein, vitamins (D, B), and long-chain omega-3 FA DHA and eicosapentaenoic acid (EPA). Individuals who consumed fish regularly had reduced risk of ischemic heart disease by 13% (283). In animal models, mice administered with fish oil diet showed reduction in platelet aggregation (284), whereas laboratory porcine fed with fish oil with PUFA showed inhibition of the synthesis of platelets thromboxane B_2_, aiding in the prevention of microthrombosis (285). Vitamins such as folic acid, B_12_ and B_6_ had been associated with a reduced risk of cerebrovascular disease such cerebral ischemia (286), whereas lower vitamin B_12_ intake had been associated with increased proportion of periventricular WMHs (287). Fish long-chain omega-3 PUFA helped to protect against vascular risk factors such as inflammation, endothelial dysfunction (with reduced circulating markers such as VCAM-1, E-selectin, and ICAM-1), and vascular resistance (i.e., improve flow-mediated arterial dilation) (288). DHA and EPA consumption had been reported to elevate PAI-1 in healthy individuals (289) and reduced the risk of RSBI and WMHs in older adults (290), while long-chain omega-3 PUFA supplementation in arteriothrombotic patients reduced the activation of prothrombin and increased TFPI (291, 292). Moreover, EPA and polyphenols helped to reduce the endogenous thrombin alongside TFPI and vitamin K–dependent factors (i.e., factors II, VII, IX, and X) and platelet aggregation, hence reducing thrombogenesis (251, 265). In addition, polyphenols helped to reduce leukocyte activation molecules such as NF-κβ and inflammatory adhesion molecules (293), ADP or collagen-mediated platelet aggregation and platelets–monocytes aggregation; reduce expression of P-selectin on platelets; and increase the release of platelet-derived NO (294).

Moreover, nuts had been reported to protect against the risk of hypertension (236) and T2DM (295), lowering cardiovascular risk, but surprisingly not against stroke (236, 260, 296). Nuts elevated the expression of TFPI in monocytes (280) and reduced TF-bearing PDMPs (297). A recent animal study revealed that mice with HFD supplemented with nuts (with high PUFA) showed a reduced plasma prothrombin level and expression of CD36 on atherosclerotic plaques in aortic region (298). Furthermore, legume (highly soluble fiber) consumptions also reported to reduce the risk of developing vascular disease, i.e., improve cholesterol level, lower GI, blood pressure, CRP, E-selectin, IL-6, TNF-α, VCAM-1, ICAM-1, and waist circumference and prevented T2DM (299–301). Previous study had reported that legumes possessed anti-inflammatory bioactive components such as inulin and oligofructose and modulated metabolic endotoxemia (302), whereas *in vivo* study showed that their secondary metabolites interacted with GIT microbiota to aid in modulating platelets hyperreactivity and potential thrombosis through the synthesis of trymethilamine N-oxide (303). A recent PREDIMED study had strengthened the fact that MeDiet possessed anti-inflammatory effects with reduced expressions of leukocyte adhesion molecules, VCAM-1, ICAM-1, reduced plasma levels of P- and E-selectin, proinflammatory cytokines (i.e., IL-1, IL-6, IL-8, CRP, TNF-α), MMPs, and chemokines (i.e., MCP-1, MIP-1) (304).

### Dietary Approaches to Stop Hypertension Diet

Dietary Approaches to Stop Hypertension (DASH) diet is a dietary pattern that encourages reduction of sodium intake (2,300 mg or 1 teaspoon per day), SFA, red and processed meat, and sweet beverages and hence characterized as diet with high intake of vegetables, legumes, fruits whole grains, nuts, low-fat dairy, fish, lean meats, and poultry (305). An increased sodium (i.e., table salts, salt additives) intake beyond the physiological requirement (high sodium-to-potassium ratio) has been shown to elevate blood pressure (306), raising the risk toward vascular disease and mortality (307). Moreover, recent systematic review had reported that an increased intake of dietary salts may increase the risk toward WMHs and ischemic stroke, i.e., lacunar stroke, and CMBs (308, 309). Previous studies had shown that DASH diet lowered the risk of developing and progression of metabolic syndrome up to 81% (310, 311), coronary heart disease by 20%, stroke by 29% (312), and the overall mortality (313). Moreover, DASH diet has been associated with improved endothelial function (314), body weight (315), inflammation grade (305), and GIT microbiota (316).

Multiple studies had also reported that DASH diet has high anti-inflammatory properties. In cross-sectional study of elderly individuals (aged 50–69 years) by Phillips and colleagues, DASH diet improved the measurement of adiposity (i.e., reduced BMI and reduced waist circumference) and lipoprotein and reduced proinflammatory, prothrombotic, and proatherogenic markers (i.e., IL-6, CRP, TNF-α, PAI-1, and leukocytes) (317). Another study showed improvements of obesogenic inflammatory markers such as reduced CRP, IL-6, and soluble ICAM-1 following DASH diet (318). A recent review also supported DASH diet beneficial effects in reducing the risk toward cancer such as breast and colorectal cancer (319). Collectively, many of these beneficial effects of DASH diet are attributable to its high-vegetable and high-fruit content, with a desirable risk reduction toward systemic and cardiovascular disease, including CSVD.

### Gluten-Free Diet

Gluten (or *glue* in Latin) refers to a group of proteins mainly found in grains such as barley, wheat, spelt, and rye. Gluten added the sticky textures and consistency to the flour once mixed with water. Glutenin and gliadin are the major examples of gluten protein reported to cause a series of ill-health effects especially in individuals with celiac disease (CD) and gluten allergy or intolerance (320, 321). The consumption of gluten-containing diet has been linked with GIT dysbiosis and leakage and gluten-induced inflammation that can lead to pathogenesis of neurodegeneration (230, 322). Moreover, high-gluten diet also elevated the proinflammatory markers in young healthy individuals (323), and there was an increased rate of superoxide and nitrotyrosine synthesis in aortic root lesion of mice model (324). A high-gluten diet also has been linked with reduced expressions of anti-inflammatory and antidysbiotic genes such as *PPAR-*γ (in intestine, peripheral inflammation, and neuroinflammation) especially in individuals with CD. This is supported by preclinical study using macaques that shown the downregulation of *PPAR-*γ–mediated inflammation in intestines, followed by GIT dysbiosis (325).

Thus, gluten-free diet (GFD) has been suggested to restore the expression *PPAR-*γ gene in CD individuals. Moreover, *in vitro* study has reported that GFD, i.e., the consumption of foods with phytocannabinoids (low dose and naturally available), such as delta-9-tetrahydrocannabinol, aided in direct activation of *PPAR-*γ gene expression, hence inhibiting intestinal inflammation in CD (326). A recent review reported that GFD is associated with a reduced risk of endothelial dysfunction and oxidative stress especially in CD individuals (327). Furthermore, an animal study also revealed that mice with GFD had reduced proinflammatory cytokines (Il-6 and TNF-α) (328). Hence, GFD is a promising approach to prevent GIT inflammation and dysbiosis and restores the integrity of epithelial barrier, thus indirectly influencing the prevention strategy in reducing risk toward other potential cardiocerebrovascular disease such as CSVD.

### Vegetarian Diets

Vegetarian diet is generally based on vegetables and fruits, and it is classified into four different styles, such as lactovegetarian (vegetarians with intake of dairy products but no eggs), ovovegetarians (intake of eggs but no dairy), ovolactovegetarians (no meat and fish, but consume both eggs and diary), and, lastly, vegan diet (absolute absence of all kind of animal-based food including seafood). Overall, vegetarian diet has been reported to reduce the risk of coronary heart disease and stroke and modulate GIT microbiota (329, 330). Meta-analysis of previous studies had shown the reduced risk factors that are linked to stroke, T2DM, and cardiovascular mortality with vegetarian diets (331–333). Moreover, a recent EPIC-Oxford study shows that vegetarian diets reduced the risk of ischemic heart disease by 22% compared to meat eaters, but with an elevated risk of hemorrhagic and total stroke (283).

Among the different types of vegetarian's diet, vegan diet has been proven to be beneficial for cardiocerebrovascular health (i.e., lower LDL cholesterol, triglycerides, and E-selectin) as it is rich with vitamins (except B_12_), polyphenols, MUFA, and fiber. However, a limited supply of vitamin B_12_ (followed by elevated level of plasma homocysteine) in vegan diets is associated with arterial endothelial dysfunction and elevated thickness of carotid intima media (334). Moreover, a higher level of polyphenol such as flavanols improved cardiovascular function (i.e., endothelial function) and endogenous repair mechanism (i.e., increase flow-mediated dilation, and reduced systolic blood pressure) (335), which helped to reduce proinflammatory, leukocyte adhesion molecules and NF-κβ, platelet aggregation, and an increase in the release of platelet-derived NO (293, 294, 336). The level of CRP also has been shown to decrease following vegetarian diets (i.e., unrefined plant foods) (337, 338) with an elevated circulating EPCs (339).

The consumption of onion and garlic in vegetarian diets has been reported to have antiplatelet, anticoagulant, and antithrombotic properties as they possess sulfur-rich element (especially in garlic) that is known to reduce platelet function and aggregation through inhibition of COX and lipo-oxygenase, followed by the suppression of thromboxane B_2_ production (340). In addition, an animal study had shown that administration of sesame seed whole grains in mice lowered the arterial thrombosis (341). Moreover, *in vitro* studies also showed that green beans extract, tomatoes extract, strawberry extract (dose-dependent: 0.1–1 mg/mL), garlic bolt, raw spinach, and blanched garlic inhibited the AA and ADP-mediated platelet aggregation; the synthesis of platelets thromboxane B_2_ reduced P-selectin and IL-1β levels (342–344) and thereby prevented thrombogenesis. These effects are believed mainly due to the presence of phenolic compounds (i.e., chlorogenic acid, ferulic acid, caffeic acid, and P-coumaric acid) in such vegetables (345). Of note, Framingham Heart Study Offspring Study reported that nutrients such as choline (precursor for acetylcholine, PS, and sphingomyelin) found in fruits (i.e., orange) and vegetables (i.e., broccoli) were associated with a lower WMHs load in relation to CSVD manifestation (257, 346).

## Diets and Circulating MPs—Proposing the Link With CSVD

For the past decades, research interests had grown on the relationship between dietary patterns and potential vascular disease including SVD pathomechanism such as cell activation and prothrombotic molecules release. Hence, treatment and management of cerebrocardiovascular disease risks such as modulating lifestyle habits and dietary pattern have been suggested as an important primary measure. Despite the advancement of understanding on the effects of diets on the release of endogenous circulating MPs toward major cardiovascular disease (i.e., atherosclerosis, coronary heart disease, and stroke), their relationships with the vascular integrity of microcirculation network and, in specific, and its roles in the pathogenesis of SVD (i.e., CSVD) require further deliberation. To date, there is no/limited study that had reported the direct impacts of diet-induced MP formation on NDD including AD and PD. At best, majority of previous studies focused mainly on the role of PDMPs and EDMPs, with scarce data available on other MP subpopulation, as well as their involvement in diet-based MP release, which may influence the risk and manifestation of SVD in general.

Previous studies had evaluated the role of diet-based circulating cell activation–derived MPs in healthy and disease populations. For example, Zhang and colleagues found that individuals with T2DM (major risk factor for CSVD) had a higher level of PDMPs and MDMPs (CD11b^+^) compared to healthy non-diabetic individuals who practice healthy chronic diet (i.e., oats rich in polyphenols and low GI) as reported with MeDiets, DASH, and vegetarian diets. Additionally, they found that P-selectin, TF, and fibrinogen-positive PDMPs are higher in T2DM individual without obesity (347) and likewise in individuals who practiced WPD with higher EDMPs and PDMPs. Of note, WPD has higher SFA, GI, and refined carbs with low to no omega-3 fatty acids (348). In contrast, HFD and LCD with higher SFA lead to an increase in MDMPs, PDMPs, and EDMPs (349). Hence, diet-based PDMP release, especially in T2DM individuals, may contribute to microthrombosis (through GP-Ib-IX-V receptor complex binding) and inflammation. In such instances, PDMPs with surface P-selectin, fibrinogen, and TF enable leukocytes–platelets adhesion, platelet aggregation, and coagulation, respectively, in small vessel and could be more vulnerable to an early development of arteriosclerosis and/or arteriolosclerosis and hence plausible link to CSVD manifestation. However, polyphenols (i.e., avenanthramide and phenolic alkaloid) found in oats (i.e., in DASH diet and MeDiet) possess antioxidant and anti-inflammatory properties (350), whereby avenanthramide is known to reduce the levels of PDMPs with specific surface markers through inhibition of platelet activation by scavenging the free radicals (from oxidative stress mediated activation), or as antagonist on activation receptors, hence mimicking antiplatelet agents. A recent study by Sinegre and colleagues indicated that epicatechin (a major subclass of flavanols found in cocoa and fruits) supplementation typically in vegetarian diets may reduce the production and release of PDMPs (GP-Ib^+^) and thrombin, respectively, without any impact on TF positive MPs which signified the effects of polyphenols on MP release and procoagulant status (351), which could influence the onset, progression, and even prevention of CSVD.

Moreover, individuals with high-gluten diet have been associated with higher systemic GIT-microbiota–derived MPs (230). However, polyphenols found in gluten-free black sorghum extract (BSE) also had been shown to possess an antioxidant property, which helped to reduce endothelial dysfunction, platelet activation or aggregation, and PDMP release mediated by oxidative stress (352, 353). Nignpense and colleagues reported that the consumption of BSE (with concentration no <40 g/mL), such as in GFD, MeDiet, and vegetarian diets, could reduce platelet aggregation (by 19%) and PDMPs (i.e., CD42b^+^) release (by 47%). The antioxidative properties found in BSE polyphenols enabled the inhibition of PDMPs through the process of hydrogen peroxide (H_2_O_2_) neutralization, free radical scavenging, and/or interruption with intracellular signaling responsible for PDMP release (353). It seems that BSE polyphenol is a potential candidate to attenuate the thrombogenic effect of PDMPs. Besides, polyphenols also reduced PDMP release through the inhibition of COX-1–mediated platelet activation (354), hence modulating microvascular environment to improve endothelium function. Also, as mentioned in the previous section, as MPs can be generated after a physiological shear stress, polyphenols (i.e., spironolactone) could modulate the blood flow (*via* NO release) and endothelium relaxation to enable the inhibition of shear stress–mediated MP release and reduced the blood pressure. In addition, grapeseed (i.e., proanthocyanins) extract administration (400 mg/kg) in mice was also shown to reduce the production of P-selectins bearing PDMPs, proinflammatory molecules (i.e., IL-6, IL-8, and TNF-α), and vWF and adhesion molecules, whereas it increased the expression of CD34 on ECs and vascular endothelial growth factor receptor 2, which resulted in the inhibition of thrombosis (355) and thus could be protective against the onset and/or progression of CSVD.

MeDiet has been widely studied and associated with the improvements of endothelium structure and function of different vasculature and vascular territories (i.e., peripheral, central, and small/micro vessel) (277). Marín and colleagues reported that MeDiet such as the consumption of extra virgin olive oil (EVOO) possessed the antioxidative properties that aided the reduction of free radical release and protected against oxidative stress, hence mitigating the production of circulating MPs (356). A high MUFA found in EVOO also reduced the levels of PDMPs (CD31^+^/CD42b^+^) and EDMPs (CD31^+^/CD42^−^) (349) that enabled the reduction of subendothelium microthrombogenicity (measured as percentage of microvascular endothelium covered by platelets and the modification or arterial wall, i.e., wall area reduction and foam cell count) in animal model (357). Hence, it is tempted to posit that, through MPs modulation, MeDiet could be protective against the onset and/or progression of CSVD.

In contrast, Weech and colleagues reported that high-SFA diets (i.e., WPD and HFD) elevated the levels of PDMPs (CD31^+^/CD42b^+^) and EDMPs (CD31^+^/AV^+^ and CD144^+^/CD6E^+^/AV^+^) (349). A recent preclinical study also indicated that mice treated with HFD had a higher level of MDMPs (CD36) (358). Hence, a higher level of prothrombotic diet–based MPs could potentially trigger the onset and advancement of CSVD. Interestingly, Marin and colleagues showed that MUFA (as attainable with MeDiet, DASH, and vegetarian diets) reduced the total count of EDMPs (CD31^+^/AV^+^ and CD144^+^/CD6E^+^/AV^+^) in healthy individuals (356), whereas Chiva-Blanch and colleagues reported EVOO consumption in MeDiet produced a reduced level of PDMPs (PAC-1^+^), SMCs-MPs (SMA-α^+^), and lymphocytes-derived MPs (CD3^+^/CD45^+^) released in individuals with a high risk of cardiovascular disease (359). Moreover, MeDiet (i.e., nuts consumption) from asymptomatic individual with cardiovascular risk (but no cardiovascular event) had also been shown to have lower levels of prothrombotic PDMPs (PAC-1^+^, CD61^+^, CD142^+^/CD61^+^ and CD62P^+^), EDMPs (CD146^+^) and other MP subpopulations such as LDMPs (CD63^+^ and CD11a^+^), suggesting that nuts consumption could modulate endothelial function *via* MP level regulation (297). Finally, MeDiet, i.e., the consumption of EVOO and nuts, facilitated the reduction of prothrombotic MPs (CD142^+^/AV^+^), procoagulant MPs (TF bearing) and cell activation MPs (CD11a^+^/AV^+^) (360, 361), that could be beneficial in the setting of CSVD prevention.

In relation to HFD, Heinrich and colleagues found that HFD elevated the production of PDMPs and EDMPs (348), whereas LCD (≥40 g/d) lowered EDMP (specifically CD31^+^/CD41^−^) levels (275). In contrast, the supplementation or consumption of fish oil (rich in EPA and DHA) such as in MeDiet and vegetarian diets reduced the EDMPs (CD31^+^/CD42b^−^) level, but not PDMPs (CD31^+^/CD42b^+^) in low to moderate cardiovascular risk individuals (362) with no effects on PDMPs (CD41^+^) level, especially in healthy individuals (363). These differences may be due to the fact that healthy individuals may have a lower degree of cellular activation in their systemic circulation. Besides, an intervention using low-calorie diet such as DASH diet in obese individuals has been reported to reduce the level of PDMPs (GP-Ib^+^) and LDMPs (CD11a^+^ and CD4^+^), but not RDMPs or EDMPs (364) despite the fact that the obese and overweight individuals possessed a higher baseline level of EDMPs (CD144^+^/CD42a^−^/CD45^−^) (365). Moreover, weight loss in non-diabetic individuals has been associated with reduced PDMPs (CD41^+^), suggesting that weight reduction may be independently mediating the inhibition of cell activation–mediated MP shedding (220). Furthermore, the consumption of cocoa flavonols (from cocoa drinks or natural cocoa), especially with DASH, MeDiet, and vegetarian diets, has been shown to reduce EDMPs (CD31b^+^/CD41^−^ and CD144b^+^) and EDMPs (CD42a/CD45^−^/CD144b^+^) in individuals with coronary artery disease and in young asymptomatic obese individuals, respectively (365, 366). Finally, HFD supplemented with cocoa polyphenols (400 mg/kg per day) fed to rats showed a reduction in platelet aggregation and an elevated release of NO and phosphorylation of eNOS by ECs (367).

Taken together, this evidence provides persuasive and plausible roles of MeDiet, DASH, GFD, and vegetarian diets in the regulation MP systemic release in guarding against microthrombi formation, whereas the formation of MPs with procoagulant TF and proinflammatory properties following WPD, HFD, and LCD is recognized to heighten the risk for microthrombosis and arteriosclerosis and/or arteriolosclerosis (368, 369) and hence risk for CSVD manifestations. Table 4 summarizes the role of dietary patterns, its corresponding molecular and cellular responses, underlying MP release, and putative predisposition toward CSVD.

**Table 4:** Summary of the role of dietary patterns, its molecular/cellular response, MP release, and risk predisposition toward CSVD.

**Dietary patterns**	**Molecular and cellular response**	**Diet-based MP correlates**	**Risk predisposition toward CSVD**
**Western Pattern Diets**			
• (+) SFA • (+) GI • (+) Refined carbs • (–) Omega-3 fatty acid	• (+) Proinflammatory response • (+) Oxidative stress • (+) Thrombin • (+) Vitamin K-dependent factors (i.e., factors II, VII, IX, X) and extrinsic TF pathway in coagulation cascade • (–) TFPI	• (+) PDMPs • (+) EDMPs	• (+) Risk T2DM • (+) Cardiometabolic syndrome • (+) Microthrombosis • (+) Iron-induced hypoxia • (+) Ischemic stroke
**High fat/low carbohydrate diets**			
• (+) SFA • (–) PUFA (LA and ALA) • (–) MUFA	• (+) FVIIa/VIIc and extrinsic TF pathway in coagulation cascade	• (+) MDMPs • (+) PDMPs • (+) EDMPs • (–) EDMPs (specifically CD31^+^/CD41^−^) in LCD	• (+) GIT Dysbiosis (i.e., inflammatory response) • (–) BDNF • (–) EPCs • (+) BBB damages • (+) WMHs
**Mediterranean diets**			
• (+) PUFA • (+) DHA/EPA • (+) MUFA • (+) Polyphenols • (–) GI • (+) Vitamins (folic acid, B_12_, B_6_)	• (+) Anti-inflammatory response • (+) Antioxidant (i.e., in EVOO) • (–) Prothrombotic coagulation • (–) ILs/NF-κβ/MMPs/VCAM/ICAM • (–) Vitamin K dependent factors (i.e., factors II, VII, IX and X) • (–) Platelet aggregation • (–) Platelets thromboxane B_2_ • (–) Prothrombin • (+) TFPI/PAI-1 • (+) NO	• (–) PDMPs • (–) EDMPs • (–) LDMPs • (–) SMCs-MPs • (–) Prothrombotic MPs • (–) Lymphocytes MPs	• (–) Risk of metabolic syndrome • (–) Microthrombosis • (–) Endothelial dysfunction • (+) Flow-mediated arterial dilation • (–) Risk of cerebrovascular disease • (–) Risk of ischemic heart disease • (–) Risk of RSBI and WMHs
**DASH diets**			
• (–) Sodium • (–) SFA • (+) Vegetarian diets	• (–) Proinflammatory, prothrombotic, and proatherogenic markers	• (–) PDMPs • (–) LDMPs • No effects on RDMPs and EDMPs	• (–) Risks of WMHs/CMBs/lacunar stroke/ischemic stroke • (–) Metabolic syndrome • (–) BMI and waist circumference • (+) Endothelial function • (+) GIT microbiota
**Vegetarian diets**			
• (+) PUFA • (+) DHA/EPA • (+) MUFA • (+) Polyphenols • (–) GI • (+) Vitamins (folic acid, B_6_) • (–) B_12_	• (–) LDL, triglycerides, and E-selectin • (–) Proinflammatory cytokines • (–) Leukocyte adhesion molecules • (–) NF-κβ • (–) Platelet aggregation • (+) Platelet-derived NO • (–) COX and lipo-oxygenase • (+) VEGF	• (–) EDMPs • (–) SMCs-MPs	• (–) Risk of coronary heart disease, stroke, T2DM • (+) GIT microbiota • (–) Arterial thrombosis • (+) Flow-mediated dilation • (–) Systolic blood pressure • (+) EPCs • Lower vitamin B_12_ associated with arterial endothelial dysfunction • (+) Phosphorylation of eNOS by ECs
**Gluten based**			
• (+) Gluten (glutenin/gliadin) • GFD • (+) Phytocannabinoids	• (+) Gluten-induced inflammation • (–) Expression of anti-inflammatory and antidysbiotic gene (i.e., PPAR-γ) • (+) MMPs • (+) Expression of PPAR-γ gene • (–) Oxidative stress • (–) Proinflammatory cytokines	• (+) Systemic GIT-microbiota derived MPs • (–) Systemic GIT-microbiota derived MPs	• (+) Pathogenesis of NDD • (+) GIT dysbiosis and leak • (–) Risk of endothelial dysfunction • (–) GIT inflammation and dysbiosis

*(+) represents increase/elevate/higher/modulate/activate; (–) represents decrease/lower/reduce/absence. ALA, α-linoleic acids; BBB, blood brain barrier; BDNF, brain-derived neurotrophic factor; BMI, body mass index; CD, cluster differentiation; CMBs, cerebral microbleeds; COX, cyclooxygenase; DHA, docosahexaenoic acids; ECs, endothelial cells, EDMPs, endothelial derived microparticles; eNOS, endothelial nitric oxide synthase; EPA, eicosapentaenoic acid; EPCs, endothelial progenitor cells; EVOO, extra virgin olive oil; GI, glycemic indexes; GIT, gastrointestinal tract; ICAM, intracellular adhesion molecules; ILs, interleukins; LA, linoleic acids; LCD, low carbohydrates diets; LDL, low density lipoprotein; LDMPs, leukocytes derived microparticles; MDMPs, monocytes derived microparticles; MMPs, matrix metalloproteinase proteins; MPs, microparticles; MUFA, monounsaturated fatty acids; NDD, neurodegenerative disease; NF-κβ, nuclear facto kappa β; NO, nitric oxide; PAI-1, plasminogen activator inhibitor 1; PDMPs, platelets derived microparticles; PPAR-γ, peroxisome proliferator-activated receptor γ; PUFA, polyunsaturated fatty acids; RDMPs, red blood cell derived microparticles; SFA, saturated fatty acids; SMCs, smooth muscle cells; T2DM, type 2 diabetes mellitus; TFPI, tissue factor pathway inhibitors; VCAM, vascular endothelial adhesion molecules; VEGF, vascular growth factors; WMHs, white matter hyperintensities*.

## Conclusion and Future Perspective

CSVD is a complex pathophysiologic condition that originates from small vessel (microcirculation) insults with brain parenchymal lesions that feature as both asymptomatic (silent) and symptomatic neurological manifestations as we grow older. One of the probable risk factors toward the onset and progression of CSVD is the imbalance and undesirable dietary patterns such as WPD and HFD. Although the impact of diets on cerebrocardiovascular disease in general has been widely studied, to date, studies on the effect on dietary pattern in CSVD remain largely unexplored. Scientific evidence provides crucial pertinent leads on diets such as vegetarians, GFD, and MeDiet that are rich in vegetables and fruits, with moderate intake of fish reducing the prevalence of major cerebrocardiovascular disease. This review presents the deliberations on the plausible roles of circulating MPs (produced by oxidative stress, inflammation, GIT microbiota dysbiosis, and cell activation) and suggests their role as one of the novel risk factor and cell-based biomarkers in diseases related to the brain–heart–GIT axis, with an emphasis on CSVD and subsequent related NDD. In particular, the understanding of the role of diet-based MPs and their communications with and/or *via* microcirculation in relation to CSVD manifestations would stir further interests in the current limited understanding on the natural history of CSVD, as well as an opportunity to devise novel approaches for its preventive and therapeutic strategies. Given that MPs can be produced and released from numerous microvascular beds of various organs (i.e., in CNS, heart, GIT, or kidney) and circulate through common systemic circulation to accumulate and exert their thrombogenic effects (i.e., prothrombotic, procoagulant, and proinflammatory) in the small end arteries especially in the cerebral microcirculation, this could contribute as a novel pathomechanism of CSVD, within the background of specific diet pattern as a modifiable precursor. A more concerted multidisciplinary and transdisciplinary research efforts to integrate the various aspects to advance our understanding of CSVD shall prove beneficial for the progressively aging society.

## Author Contributions

CMNCMN conceived the original idea, designed the outlines of the review, gathered the literature and resources, drafted, prepared the figures, tables and revised the manuscript. MM designed the outlines of the review, drafted, reviewed, and revised the manuscript. MMG, SH, NSI, WJH, HHN, CHG, LSY, NJ, YN, LF, LKO, HAH, HNA provided the resources, critically reviewed, revised, and improved the manuscript. All authors have read and approved the final manuscript.

## Conflict of Interest

The authors declare that the research was conducted in the absence of any commercial or financial relationships that could be construed as a potential conflict of interest.
